# Four putative SWI2/SNF2 chromatin remodelers have dual roles in regulating DNA methylation in Arabidopsis

**DOI:** 10.1038/s41421-018-0056-8

**Published:** 2018-10-16

**Authors:** Dong-Lei Yang, Guiping Zhang, Lili Wang, Jingwen Li, Dachao Xu, Cuiru Di, Kai Tang, Lan Yang, Liang Zeng, Daisuke Miki, Cheng-Guo Duan, Huiming Zhang, Jian-Kang Zhu

**Affiliations:** 1 Shanghai Center for Plant Stress Biology and Center of Excellence in Molecular Plant Sciences, Chinese Academy of Science, Shanghai, 200032 China; 2 State Key Laboratory of Crop Genetics and Germplasm Enhancement, Nanjing Agricultural University, Nanjing, 210095 China; 3 Department of Horticulture and Landscape Architecture, Purdue University, West Lafayette, 47907 IN, USA

## Abstract

DNA methylation is a conserved epigenetic mark that is critical for many biological processes in plants and mammals. In Arabidopsis, the antagonistic activities of RNA-directed DNA methylation (RdDM) and ROS1-dependent active DNA demethylation are key for the dynamic regulation of locus-specific DNA methylation. However, the molecular factors that coordinate RdDM and active demethylation are largely unknown. Here we report that CLSY4 and its three paralogous SWI2/SNF2-type chromatin-remodeling proteins function in both RdDM and DNA demethylation in Arabidopsis. We initially identified CLSY4 in a genetic screen for DNA demethylation factors and subsequently demonstrated that it also is important in RdDM. Comprehensive genetic analyses using single and high order mutants of CLSY family proteins revealed their roles as double agents in the balance between methylation and demethylation reactions. The four CLSY proteins collectively are necessary for the canonical RdDM pathway; at the same time, each CLSY likely mediates DNA demethylation at specific loci where DNA methylation depends on RdDM. These results indicate that the four chromatin-remodeling proteins have dual functions in regulating genomic DNA methylation, and thus provide new insights into the dynamic regulation of DNA methylation in a model multicellular eukaryotic organism.

## Introduction

DNA methylation is an important epigenetic mark that confers transcriptional regulation of genes and transposable elements^1–4^. Aberrant DNA methylation can disrupt normal developmental processes and cause disease symptoms in plants and mammals^5,6^. A specific DNA methylation pattern is an outcome of dynamic regulation by methylation establishment and maintenance, together with methylation removal activities.

In plants, de novo DNA methylation can be established by the RNA-directed DNA methylation (RdDM) pathway, in which complementary pairing between 24-nt siRNAs and nascent scaffold RNAs, together with protein–protein interactions, recruits the DNA methyltransferase DRM2 for DNA methylation^4,7–10^. Production of nearly all 24-nt siRNAs depends on the plant-specific RNA polymerase Pol IV, which generates single-stranded non-coding RNAs that serve as templates for downstream processing by RDR2 (RNA-dependent RNA polymerase 2) and DCL3 (Dicer-like 3) to produce 24-nt siRNAs^11–17^. Pol IV is recruited to RdDM targets in part by a SAWADEE domain-containing protein, SHH1/DTF1, which binds via its Tudor domain to histone H3 with methylated H3K9^18,19^. Both Pol IV and DTF1/SHH1 associate with CLSY1^18,19^, which is a chromatin-remodeling protein involved in Pol IV-dependent siRNA production^20^. However, it appears that CLSY1 only affects a part of Pol IV-dependent RdDM^21^, and it is unclear whether other chromatin-remodeling proteins are involved in Pol IV-dependent siRNA production and DNA methylation.

Active DNA demethylation counteracts the establishment and maintenance of DNA methylation to prevent DNA hypermethylation at specific loci^22^. In plants, a family of bifunctional 5-mC DNA glycosylases initiates active DNA demethylation through a base excision repair pathway^23–26^. ROS1 is a major DNA demethylase in Arabidopsis and is particularly important for counteracting DNA methylation established by the RdDM pathway, although it also demethylates RdDM-independent methylation at some genomic regions^22,23,27,28^. The *ROS1* gene promoter contains a short DNA sequence termed MEMS (DNA MEthylation Monitoring Sequence). DNA methylation is positively correlated with *ROS1* gene expression and is co-regulated by the RdDM pathway, by the CG methylation maintenance methyltransferase MET1, and by ROS1 itself;^29–31^ thus MEMS serves as an indicator of general methylation and demethylation activities and enables the coordination of DNA methylation and active DNA demethylation through transcriptional regulation of *ROS1*. Although it is evident that DNA methylation is dynamically regulated by methylation and demethylation processes, the underlying mechanisms of this fine dynamic regulation, besides the transcriptional regulation of *ROS1*, remain to be discovered.

In this study, our genetic screen for Arabidopsis DNA demethylation factors led to the isolation of a *clsy4* mutant. Investigations on single, triple, and quadruple mutants of CLSY1, CLSY2, CLSY3, and CLSY4 revealed that these four chromatin-remodeling proteins not only redundantly function in Pol IV-dependent RdDM, but also individually mediate active DNA demethylation at specific genomic regions including RdDM targets. Our results show that the four CLSY proteins collectively are necessary for Pol IV-dependent siRNA production and DNA methylation in the canonical RdDM pathway. Importantly, each CLSY also mediates DNA demethylation at loci where DNA methylation depends on one or more of the other three CLSY proteins, thereby providing a novel mechanism underlying the dynamic regulation of DNA methylation in Arabidopsis.

## Results

### Identification of CLSY4/CHR40 as a DNA demethylation factor

**Fig. 1:**
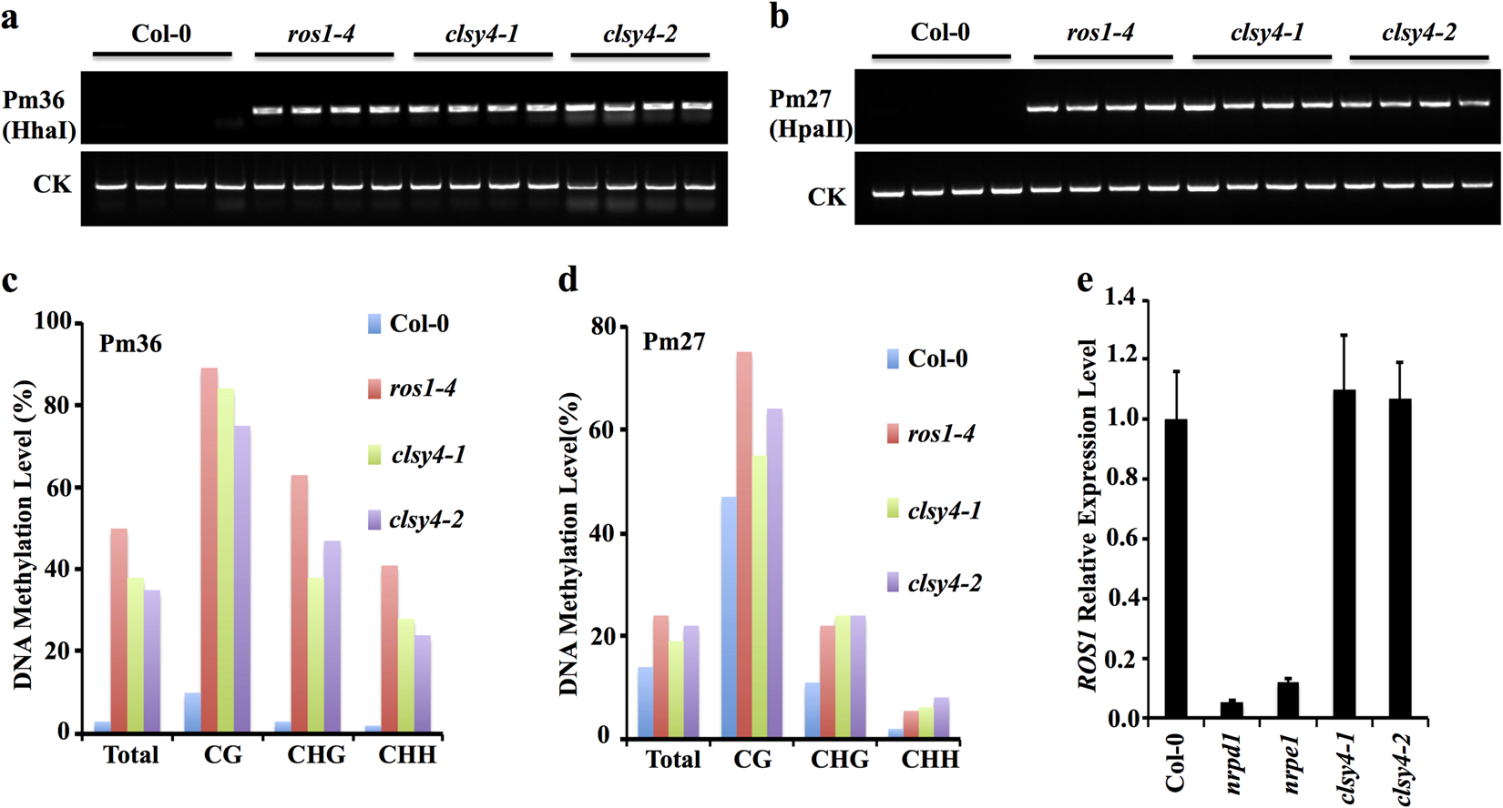
Identification of CLSY4/CHR40 as a regulator of DNA demethylation. **a** DNA methylation analysis at Pm36 by chop-PCR with digestion by the methylation-sensitive enzyme HhaI. Undigested DNA was used as the control (CK). **b** DNA methylation analysis at Pm27 by chop-PCR with digestion by the methylation-sensitive enzyme HpaII. Undigested DNA was used as the control. **c** and **d** Individual locus bisulfite sequencing at Pm36 (**c**) and Pm27 (**d**). **e** qRT-PCR analysis of *ROS1* transcript levels. Bars indicate the standard error of three biological replicates

In searching for molecular factors involved in active DNA demethylation, we conducted a genetic screen in a T-DNA mutant collection (Supplementary Table S1) using Chop-PCR to assess the DNA methylation status of two genomic loci, Pm36 and Pm27 (Supplementary Fig. S1a). Pm36 and Pm27 are located in the 3′ regions of At1g26400 and At1g26390, respectively, and both show DNA hypermethylation in the *ros1* mutant and were used successfully as markers to isolate *idm1* (*increased DNA methylation 1*) and *idm2* (*increased DNA methylation 2*) mutants that are defective in the regulation of active DNA demethylation (Supplementary Fig. S1a)^32,33^. In a T-DNA insertion mutant (SALK_102252) of At3g24340, both Pm36 and Pm27 could be amplified after methylation-sensitive enzyme digestion, suggesting DNA hypermethylation in this mutant (Fig. 1a, b; Supplementary Fig. S1b, c). Moreover, another T-DNA insertion mutant allele for this gene (SALK_003876) also exhibited DNA hypermethylation (Fig.1a, b; Supplementary Fig. S1b, c). At3g24340 encodes Chromatin Remodeling 40 (CHR40), a member of the ATP-dependent chromatin remodeler protein family that is also known as CLSY4^34^. To confirm the DNA hypermethylation phenotype, we performed bisulfite sequencing at the two loci and found that DNA methylation levels in all cytosine contexts (CG, CHG, and CHH, where H represents A, T, and C) were increased in both *clsy4/chr40* alleles as is also the case in the *ros1* mutant (Fig. 1c, d).

Previous reports showed that the reduced *ROS1* expression in various RdDM mutants causes increased DNA methylation at certain loci in the genome^22,30,35^. Given that CHR40 was previously found to be associated with the Pol IV protein complex^34^, we suspected that DNA hypermethylation in *clsy4/chr40* mutants might be caused indirectly by a decrease in *ROS1* expression. However, real-time PCR assays showed that the transcript level of *ROS1* was not changed in either of the *clsy4/chr40* mutant alleles compared to wild-type plants (Fig. 1e), suggesting that CLSY4/CHR40 is more likely to be directly involved in the ROS1-mediated active DNA demethylation pathway.

In addition, transgenic expression of *CLSY4* driven by its native promoter rescued the CHH and CHG hypermethylation phenotype in the *clsy4-1*/*chr40-1* mutant (Supplementary Fig. S1d, e), supporting the inference that the mutations in *CLSY4/CHR40* are responsible for DNA hypermethylation at the tested loci.

### CLSY4 affects DNA methylation at many genomic regions

**Fig. 2:**
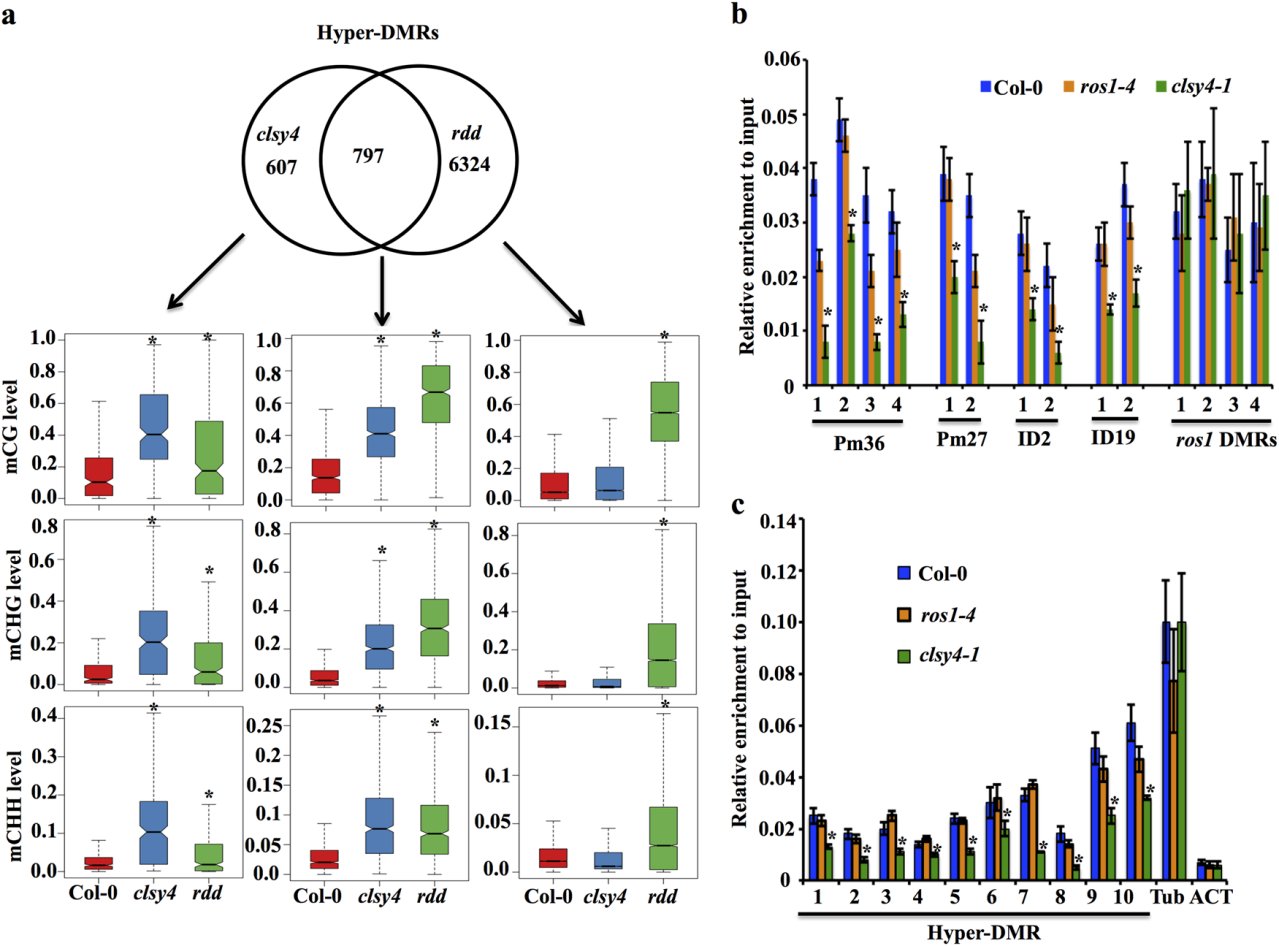
Features of *clsy4* hyper-DMRs. **a** Venn diagram showing the overlap of hyper-DMRs between *clsy4* and *rdd* mutants, and box plots showing the DNA methylation levels in three sequence contexts at *clsy4-*specific, *rdd-*specific, and overlapped DMRs. *Indicates significant difference by Wilcoxon sum test (*P* < 0.01). **b** Nucleosome density indicated by histone H3 levels at the Pm36, Pm27, ID2, and ID19 loci with four *rdd*-specific hyper-DMRs as controls measured by chromatin immunoprecipitation (ChIP). Bars indicate the standard deviation of three biological replicates. *Indicates statistical difference between the wild type and *clsy4* according to Student’s *t*-test (*P* < 0.01). **c** Nucleosome density indicated by histone H3 levels at 10 *clsy4* hyper-DMRs measured by ChIP. Tubulin 8 and actin 2 served as controls. *Indicates statistical difference between the wild type and *clsy4* according to Student’s *t*-test (*P* < 0.01). Bars indicate the standard deviation of three biological replicates

CLSY4/CHR40 belongs to the SWI2/SNF2 family of chromatin-remodeling proteins, and is in the clade of six plant-specific members (Supplementary Fig. S2a) that include CLSY1 and DRD1; DRD1 is a component of the DDR complex required for Pol V function in RdDM^36^. A previous study reported that CLSY4 could be co-purified with the largest subunit of Pol IV, suggesting its possible role in RdDM^34^. However, our results above suggested that CLSY4 functions in DNA demethylation. To investigate this inconsistency, we performed high coverage (~60x) whole-genome bisulfite sequencing of the *clsy4-1*/*chr40-1* mutant in order to examine the DNA methylome of *clsy4* mutant plants (Supplementary Table S2). We identified 1404 hypermethylated differentially methylated regions (hyper-DMRs) and 314 hypomethylated DMRs (hypo-DMRs) in *clsy4-1* mutant plants compared to wild-type plants (Supplementary Fig. S2b). For the hyper-DMRs, DNA methylation in three sequence contexts, including CG, CHG, and CHH, was increased in the *clsy4-1*/*chr40-1* mutant (Fig. 2a). This pattern of increased methylation in all three sequence contexts was also observed in *ros1* single and *ros1dml2dml3* (*rdd*) triple mutants^32^. According to our analysis, about 60% of the hyper-DMRs in *clsy4-1/chr40-1* also showed hypermethylation in the *rdd* mutant (Fig. 2a). Even for the *clsy4*-specific hyper-DMRs, DNA methylation in CG, CHG, and CHH contexts was also significantly increased in *rdd* mutant plants, although the increase was less than that in *clsy4* (Fig. 2a), possibly due to the remaining 5-mC DNA glycosylase DME in *rdd*.

Increased DNA methylation levels at two hyper-DMRs, ID2 (increased DNA methylation 2) and ID19, were confirmed by individual locus bisulfite sequencing (Supplementary Fig. S2c, d). The siRNA abundance at Pm36, ID2, and ID19 was not increased in *clsy4* mutants, as shown by northern blotting (Supplementary Fig. S2e), indicating that the DNA hypermethylation was not due to increased accumulation of siRNAs. Given that *CLSY4* encodes a putative ATP-dependent chromatin-remodeling protein that might regulate nucleosome positioning, we assessed the nucleosome occupancy at the hyper-DMRs^37^ using anti-histone H3 for ChIP-qPCR assays after MNase digestion. At four genomic regions (Supplementary Fig. S3a) that were confirmed to be hypermethylated by individual locus bisulfite sequencing in both *clsy4* and *ros1* (Fig. 1c, d; Supplementary Fig. S2c, d), the ChIP signals were significantly lower in *clsy4* mutant plants than in wild-type plants, indicating that the nucleosome occupancy at those regions was reduced in *clsy4* (Fig. 2b). In contrast, the signals in the wild type and the *ros1* mutant were comparable (Fig. 2b), indicating that ROS1 does not affect nucleosome positioning. In addition, at four *rdd* mutant-specific hyper-DMRs (Supplementary Fig. S3b), the nucleosome occupancy was not deceased in the *clsy4* mutant (Fig. 2b), suggesting a direct role of CLSY4 in the regulation of active demethylation. At 10 other randomly selected hyper-DMRs of *clsy4* (Supplementary Fig. S4), the *clsy4* mutant also showed lower nucleosome occupancy than the wild type and *ros1* (Fig. 2c). These results suggest that CLSY4 likely facilitates active DNA demethylation through nucleosome remodeling.

To determine whether the increased DNA methylation in *clsy4* affects gene expression, we selected 12 genes near the hyper-DMRs and measured their expression levels (Supplementary Figs. S5, S6). Eight of the 12 selected genes showed reduced transcript levels in the *clsy4* mutant (Supplementary Figs. S5, S6). One gene, At3g44910, showed an increased expression in *clsy4* and *ros1* (Supplementary Fig. S6a), providing a case in which DNA methylation is positively correlated with the transcript level. Taken together, the results show that CLSY4-dependent DNA demethylation contributes to gene regulation.

**Fig. 3:**
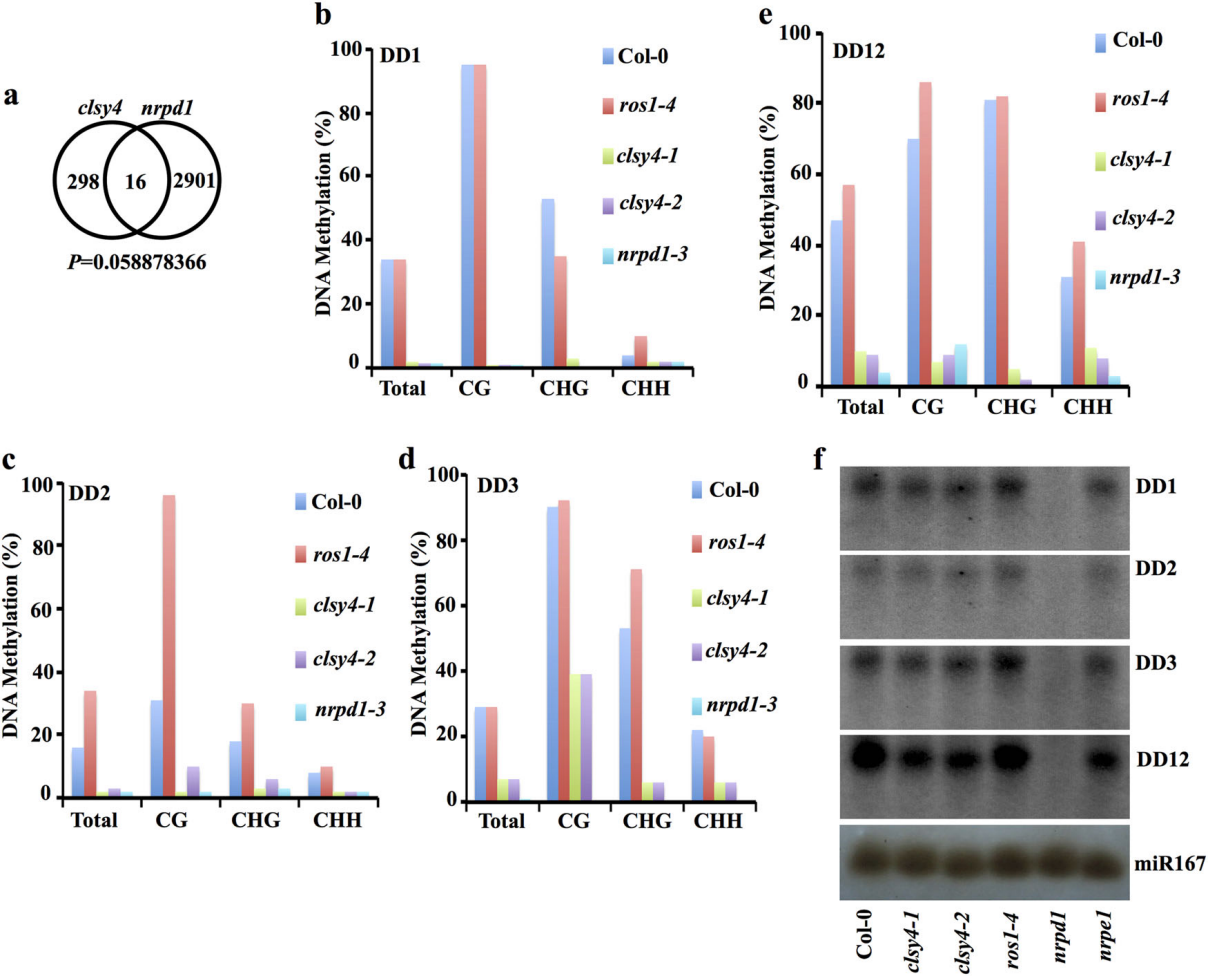
CLSY4 is required for DNA methylation at certain RdDM target loci. **a** Venn diagram showing the overlap of hypo-DMRs between *clsy4* and *nrpd1*. **b**–**e** The DNA methylation levels at the DD1, DD2, DD3, and DD12 loci as determined by individual locus bisulfite sequencing. **f** Small RNA northern blots showing siRNA abundance at the indicated hypo-DMRs

Among the 314 hypo-DMRs identified in the *clsy4-1*/*chr40-1* mutant, only 16 overlapped with *nrpd1* hypo-DMRs, suggesting that a large subset of the hypo-DMRs in *clys4* mutant are not RdDM target loci (Fig. 3a). Four common hypo-DMRs were selected for validation by individual locus bisulfite sequencing, which showed that DNA methylation was indeed reduced in both the *clsy4* and *nrpd1* mutants relative to the Col-0 wild type and *ros1* mutant (Fig. 3b–e). siRNAs were abolished at the tested loci in the *nrpd1* mutant and were not reduced in the *clsy4* mutant (Fig. 3f). siRNA accumulation at several known RdDM target loci such as Solo-LTR, AtSN1, and GP1, was unaffected in *clsy4* (Supplementary Fig. S7a); DNA methylation at these loci was also unaffected in *clsy4* mutant plants (Supplementary Fig. S7b–d). Altogether, our genome-wide analyses of DMRs in *clsy4* revealed that CLSY4 is likely involved in DNA demethylation and, to a lesser degree, in Pol IV-dependent RdDM.

### The relationship between RdDM and DNA demethylation mediated by CLSY4

**Fig. 4:**
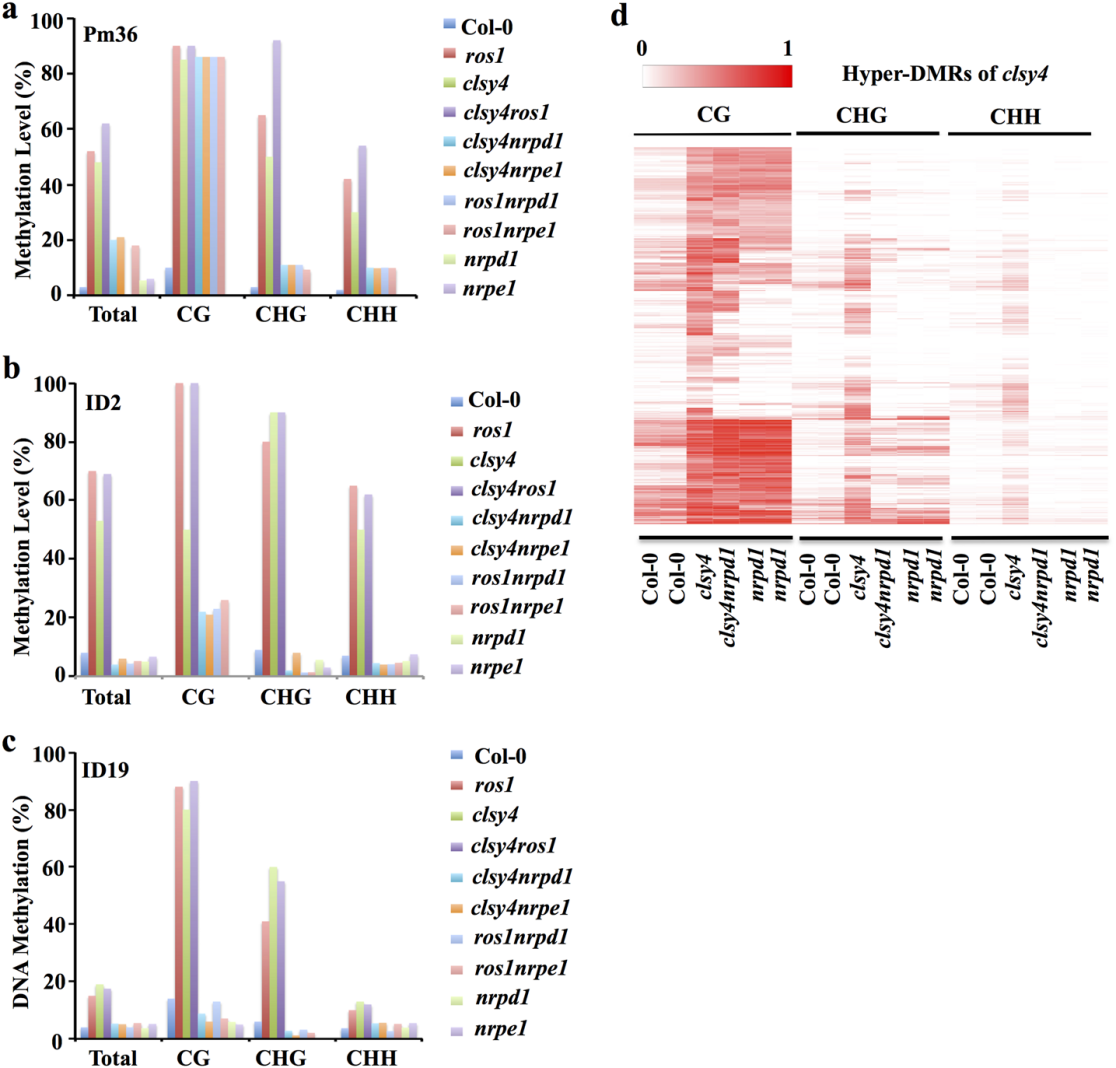
DNA hypermethylation of *clsy4* depends on RdDM. **a**–**c** DNA methylation levels at the indicated *clsy4* hyper-DMRs in various genotypes as measured by individual locus bisulfite sequencing. **d** Heatmap analysis of the DNA methylation levels of three types (CG, CHG, and CHH) of *clsy4* hyper-DMRs in the indicated genotypes

To investigate the relationship between CLSY4-mediated DNA demethylation and RdDM, we carried out genetic analysis in the *clsy4-1nrpd1-3* and *clsy4-1nrpe1-11* double mutants at three *clsy4* hyper-DMRs (Pm36, ID2, and ID19). These tested loci are also ROS1 target loci (Fig. 4a–c). At these loci, the DNA methylation in CHG and CHH contexts was substantially lower in the double mutants *clsy4nrpd1, clsy4nrpe1*, *ros1nrpd1*, and *ros1nrpe1* than that in the *clsy4* and *ros1* mutants (Fig. 4a–c), indicating that these loci are RdDM targets. The variation in CG methylation was substantial and included full maintenance at PM36 in *clsy4nrpd1*, *clsy4nrpe1*, *ros1nrpd1*, and *ros1nrpe1*; partial maintenance at ID2 in *ros1nrpd1* and *ros1nrpe1*; and complete reduction at ID19 in *clsy4nrpd1*, *clsy4nrpe1*, *ros1nrpd1*, and *ros1nrpe1* (Fig. 4a–c). These results suggest that the DNA hypermethylation in *clsy4* depends on the RdDM pathway. We did not observe any additive effect between *clsy4* and *ros1* by comparing the DNA methylation levels at these loci in *clsy4* and *ros1* single mutants to those in the *ros1clsy4* double mutant (Fig. 4a–c). These genetic analysis results, together with the data showing that the DNA methylation levels at *clsy4* hyper-DMRs were increased in *rdd* (Fig. 2a), strongly suggest that CLSY4/CHR40 and the ROS1 family DNA demethylases function in the same genetic pathway for DNA demethylation.

To further examine the dependence of the *clsy4* hyper-DMRs on RdDM, we conducted high coverage (~58x) methylome analysis of the *clsy4nrpd1* double mutant using whole-genome bisulfite sequencing (Supplementary Table S2). The cytosine methylation level at *clsy4* hyper-DMRs was clearly reduced in the *clsy4nrpd1* double mutant (Supplementary Fig. 8). In the CHG and CHH sequence contexts, DNA methylation in the double mutant was significantly lower than that in the Col-0 wild type, but was comparable to that in the *nrpd1* mutant, whereas DNA methylation in the CG context was only slightly reduced in the double mutant (Supplementary Fig. S8a–d). Heatmap analysis also showed that the CHH methylation at most *clsy4* hyper-DMRs was decreased in the *clsy4nrpd1* double mutant (Fig. 4d), while CHG methylation was reduced at the majority of the hyper-DMRs, and CG methylation was reduced at a subset of the hyper-DMRs in the double mutant (Fig. 4d). These results suggest that the hypermethylation of non-CG sequences throughout the genome in *clsy4* depends on RdDM.

### Four CLSYs redundantly regulate DNA methylation at RdDM target loci

**Fig. 5:**
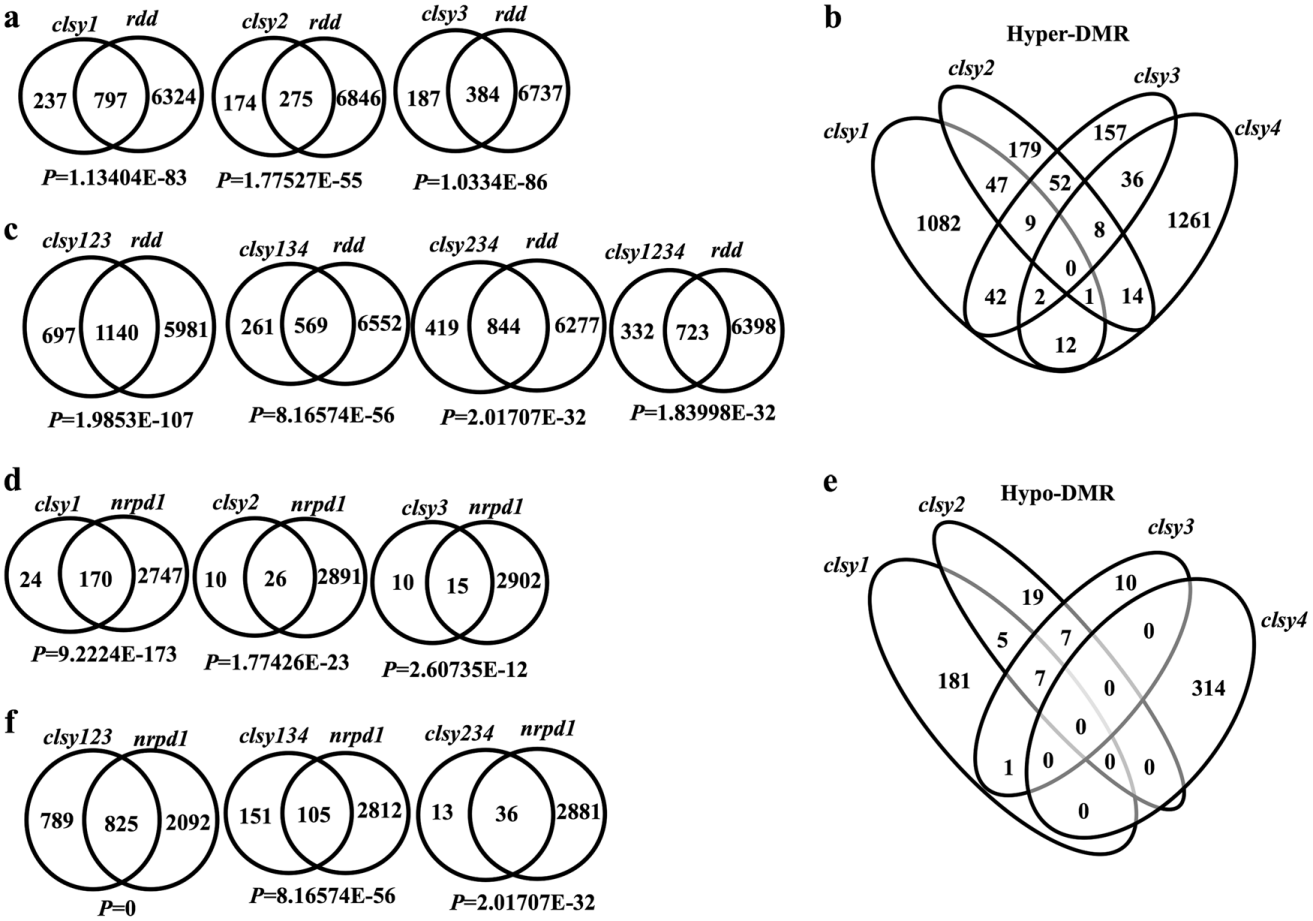
Analyses of DMRs in *clsy* single and triple mutants. Venn diagram showing the overlap of hyper-DMRs between **a** *rdd* and *clsy1*, *clsy2*, or *clsy3*; **b** *clsy* single mutants; and **c** *rdd* and *clsy123*, *clsy134*, *clsy234*, or *clsy1234*. Venn diagram showing the overlap of hypo-DMRs between **d** *nrpd1* and *clsy1*, *clsy2*, and *clsy3*; **e** *clsy* single mutants; and **f**. *nrpd1* and *clsy123*, *clsy134*, and *clsy234*. *P*-values are indicated under each overlap (hypergeometric distribution test)

The Arabidopsis CLSY1, CLSY2, CLSY3, and CLSY4 are four closely related chromatin-remodeling proteins and thus may have redundant functions in regulating DNA methylation. To understand their genetic relationships, we crossed their respective mutants and constructed high order mutants including three triple mutants (*clsy123* refers to *clsy1clsy2clsy3*, *clsy134* refers to *clsy1clsy3clsy4*, and *clsy234* refers to *clsy2clsy3clsy4*) and a quadruple mutant (*clsy1234* refers to *clsy1clsy2clsy3clsy4*) (Supplementary Table S3). Whole-genome bisulfite sequencing was conducted in the high order mutants together with the single mutants with high coverage (Supplementary Table S2). Hundreds of hyper-DMRs were identified in *clsy1*, *clsy2*, and *clsy3* single mutants, with the majority being shared by the *rdd* mutant, i.e., 77% in *clsy1*, 61% in *clsy2*, and 67% in *clsy3* overlapped with the hyper-DMRs in *rdd* (Fig. 5a). Consistent with the hypermethylation in *clsy* single mutants, the CHG and CHH methylation level of transposons was higher in *clsy1*, *clsy2*, and *clsy3* than that in the wild type (Supplementary Fig. S9a). The overlapped hyper-DMRs among the four *clsy* single mutants were very limited (Fig. 5b), suggesting that each CLSY controls DNA demethylation in a locus-specific manner. The majority of the hyper-DMRs in the triple mutants and the quadruple mutant were also shared by *rdd*, with 62% overlap for *clsy123*, 69% overlap for *clsy134*, 67% overlap for *clsy234*, and 69% overlap for *clsy1234* (Fig. 5c).

In contrast to the hypo-DMRs in *clsy4* (Fig. 3a), the majority of hypo-DMRs in the other three *clsy* single mutants were shared by *nrpd1* (Fig. 5d), indicating important roles of CLSY1, 2, and 3 in RdDM. Again, each CLSY affected DNA methylation in largely distinct genomic regions (Fig. 5e). Even though certain hypo-DMRs were identified in the four *clsy* single mutants, DNA methylation levels at gene body and transposon regions in the single mutants were not as low as those in the *nrpd1* mutant (Supplementary Fig. S9a), suggesting that the four CLSYs are largely redundant in mediating RdDM. The triple mutant *clsy123* had the highest number of hypo-DMRs (*n* = 1614), while *clsy134* had an intermediate number (*n* = 256), and *clsy234* had the lowest number (*n* = 49) (Fig. 5f). The majority of the hypo-DMRs in the three triple mutants were RdDM targets (Fig. 5f). In addition, among the three triple mutants, *clsy123* had the lowest and *clsy234* had the highest CHH methylation levels at both gene and transposon regions (Supplementary Fig. S9b), suggesting a predominant role of CLSY1 and a minor role of CLSY4 in controlling of DNA methylation at RdDM target regions.

**Fig. 6:**
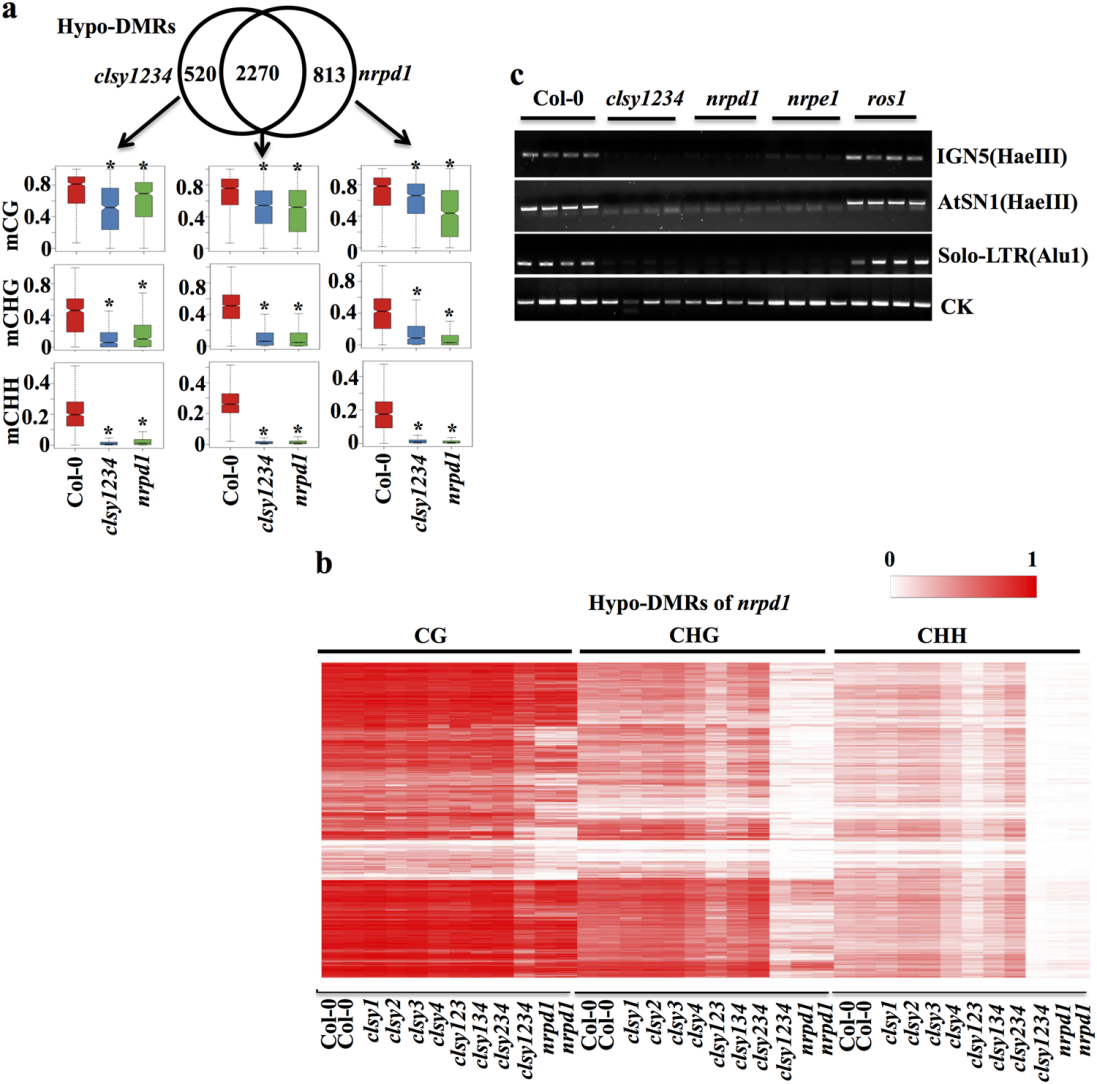
Simultaneous mutations in all four CLSYs eliminate DNA methylation at RdDM target loci. **a** Methylation levels of CG, CHG, and CHH at *clsy1234-*specific, *nrpd1-*specific, and their common hypo-DMRs. **b** Heatmap showing the methylation levels in various *clsy* mutants at *nprd1* hypo-DMRs in different cytosine contexts (CG, CHG, and CHH). **c** Analysis of DNA methylation in the indicated genotypes at IGN5, AtSN1, and Solo-LTR by chop-PCR. Undigested DNA served as the control

The CHG and CHH methylation levels at gene body regions in the *clsy1234* mutant were quite similar to those in *nrpd1* (Supplementary Fig. S9b). At transposons, which are the major RdDM targets, the CHG and CHH methylation levels were reduced more in *clsy1234* than in *nrpd1* (Supplementary Fig. S9b). Thousands of hypo-DMRs were identified in *clsy1234*, the majority of which were shared by *nrpd1* (Fig. 6a). Importantly, the methylation level was also reduced in *nrpd1* at *clsy1234-*specific hypo-DMRs, and vice versa (Fig. 6a). Heatmap analysis showed highly similar patterns of CHG and CHH hypomethylation in *clsy1234* and *nrpd1* at *nrpd1* hypo-DMRs (Fig. 6b). DNA hypomethylation at RdDM target loci was observed in the single mutants *clsy1* and *clsy4*, whereas *clsy2* and *clsy3* showed little difference compared to the wild type (Fig. 6b). Non-CG methylation was moderately reduced in *clsy123* and was less reduced in *clsy134*, whereas *clsy234* showed little reduction in non-CG methylation (Fig. 6b). The methylation analyses of RdDM regions of the single and triple mutants also suggested that CLSY1 predominates among the four CLSY proteins in mediating RdDM, which might explain why CLSY1 but not the other three CLSY proteins has been identified from genetic screens for RdDM factors^20^. These results demonstrate that the four CLSY members redundantly control DNA methylation at RdDM target loci. DNA methylation at several known RdDM target loci was also examined using chop-PCR. The chop-PCR results showed that IGN5, AtSN1, and Solo-LTR were hypomethylated in *clsy1234* as well as in the RdDM mutants *nrpd1* and *nrpe1* (Fig. 6c), supporting the inference that RdDM was blocked in the quadruple mutant.

### *clsy* mutations impair siRNA production and transposon silencing

**Fig. 7:**
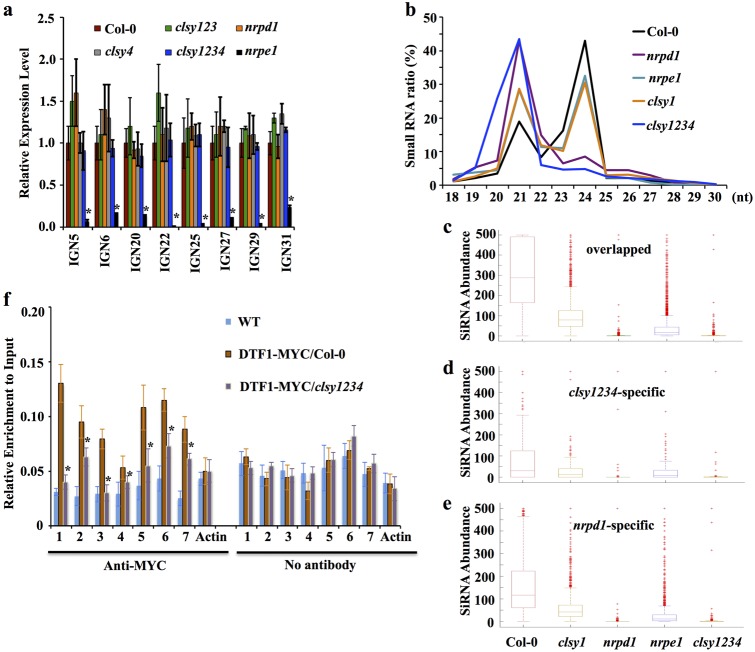
Pol V transcripts, small RNA abundance, and DTF1/SHH1 enrichment in the *clsy1234* mutant. **a** Pol V transcript levels in the indicated genotypes. **b** Length distribution of small RNAs in the indicated genotypes. **c** siRNA abundance at *clsy1234* and *nrpd1* overlapped hypo-DMRs. **d** siRNA abundance at *clsy1234-*specific hypo-DMRs. **e** siRNA abundance at *nrpd1-*specific hypo-DMRs. **f** Relative ChIP signals for DTF1/SHH1 in the indicated genotypes. *Indicates statistical difference between DTF1-MYC/Col-0 and DTF1-MYC*/clsy1234* according to Student’s *t*-test (*P* < 0.01)

Because DRD1 is a putative chromatin-remodeling protein that is closely related to the CLSYs and that functions to facilitate Pol V transcription^36^, we tested whether the CLSY proteins might also be involved in Pol V transcription to produce the scaffold RNAs essential for AGO4 targeting^38^. Quantitative RT-PCR assays showed that the transcript levels of eight Pol V scaffold RNAs in *clsy4*, *clsy123*, and *clsy1234* were comparable to those in the wild type (Fig. 7a), suggesting that the CLSYs are not involved in Pol V transcription. The four CLSYs proteins are physically associated with Pol IV, which indicates their potential roles in siRNA biosynthesis^34^. To test this possibility, we conducted small RNA sequencing using *clsy1234* mutant plants. As was the case in *nrpd1*, 24-nt siRNAs were almost eliminated in the quadruple mutant (Fig. 7b). In the *clsy1* single mutant, 24-nt siRNAs were slightly reduced, resembling the *nrpe1* mutant (Fig. 7b). These patterns suggest that the four CLSYs are collectively required for 24-nt siRNA production. At the *clsy1234-*specific, *nrpd1-*specific, and their overlapped hypo-DMRs (Fig. 6a), 24-nt siRNAs were nearly eliminated in *clsy1234* and *nrpd1* mutants but were moderately reduced in *clsy1* and *nrpe1* mutants (Fig. 7c–e), supporting the inference that the four CLSYs redundantly regulate siRNA biogenesis at all RdDM target loci. Because DTF1/SHH1 is required for Pol IV function and 24-nt siRNA biogenesis^18,19^, we examined DTF1/SHH1 enrichment at several RdDM target loci (Supplementary Fig. S10) and found that the chromatin occupancy of DTF1/SHH1 was less in *clsy1234* plants than that in wild-type plants (Fig. 7f). This suggested that the four CLSYs are required for proper DTF1/SHH1 binding at the RdDM target loci.

**Fig. 8:**
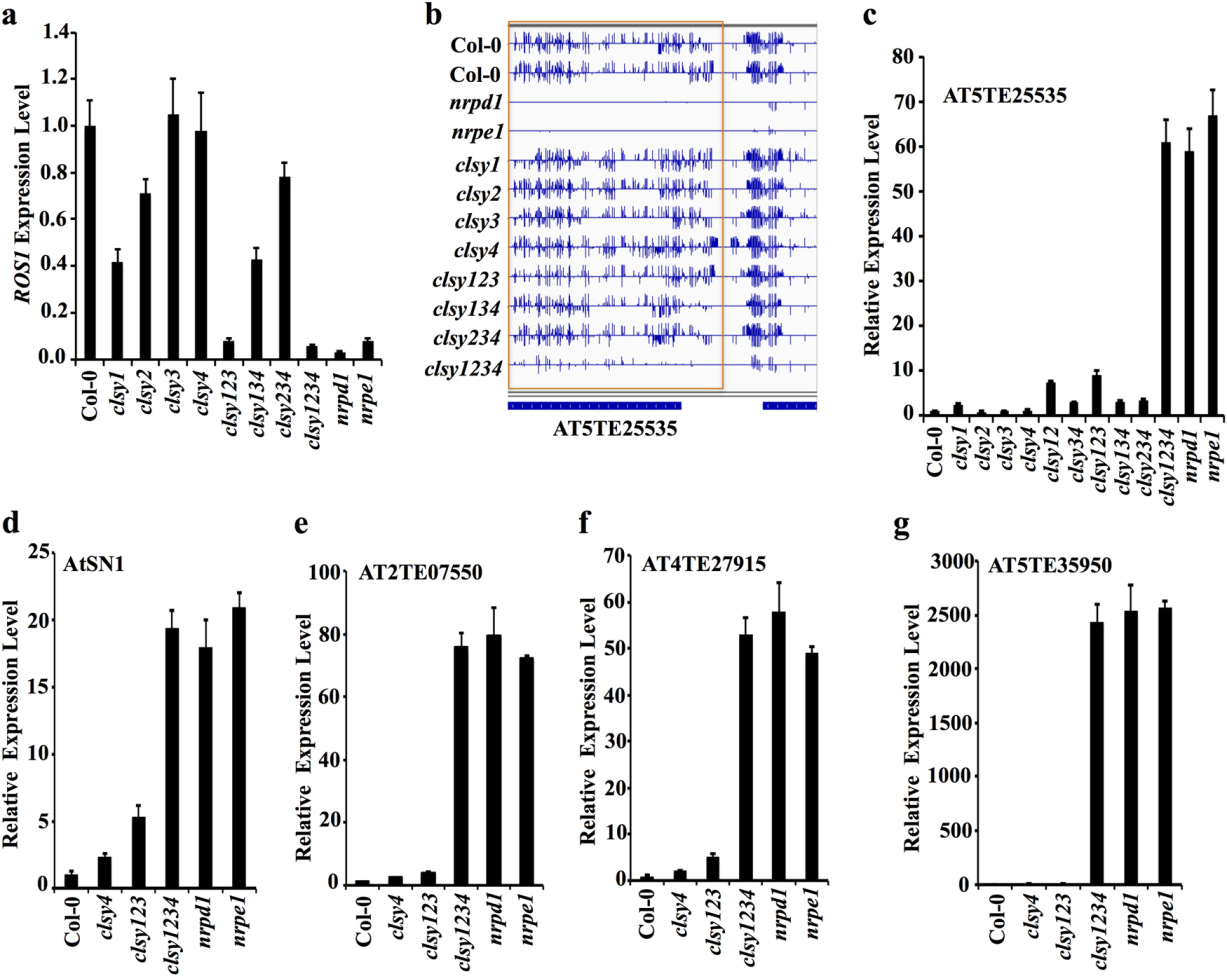
The four CLSYs redundantly regulate transposon expression. **a** Real-time PCR assay for *ROS1* transcript levels in various *clsy* mutants. **b** Snapshot showing the DNA methylation of AT5TE25535 in various genotypes. **c** Real-time PCR assay for AT5TE25535 expression levels in various *clsy* mutants. **d**–**g** The expression levels of the indicated transposons in various mutants

*ROS1* expression level is an excellent indicator ofo RdDM activities, because *ROS1* expression positively correlates with RdDM-dependent DNA methylation in its promoter region^30,39^. Like *nrpd1* and *npre1*, *clsy1234* showed a drastic reduction in *ROS1* transcript levels (Fig. 8a). Among the four single *clsy* mutants, *clsy1* had the lowest *ROS1* expression, while *clsy123* had the lowest *ROS1* expression among the three triple mutants (Fig. 8a). These patterns of *ROS1* gene repression are consistent with the DNA hypomethylation phenotypes in the mutants (Fig. 6b).

Because RdDM can transcriptionally silence transposons, we selected several TEs that are known to be released in the *drd1* mutant^40^, and examined their DNA methylation levels and transcript levels in various *clsy* mutants and *nrpd1* and *nrpe1* mutants. As shown in Fig. 8b, DNA methylation at AT5TE25535 was nearly eliminated in *clsy1234* but not in the other *clsy* mutants (Fig. 8b). Consistent with the DNA methylation patterns, the expression level of AT5TE25535 in *clsy1234* was increased by about 60 fold, which was comparable to that in *nrpd1* and *nrpe1* mutants; in the other *clsy* mutants, in contrast, AT5TE25535 expression levels were either not changed or were only slightly increased (Fig. 8c). As was the case at AT5TE25535, DNA methylation at the other four tested transposons was eliminated in *clsy1234*, *nrpd1*, and *nrpe1* mutants, but remained largely unchanged in the other *clsy* mutants (Supplementary Fig. S11a–d). Their transcript levels were dramatically increased in *clsy1234* as well as in *nrpd1* and *nrpe1* (Fig. 8d–g). Therefore, consistent with their redundant roles in RdDM, the four CLSY proteins have redundant roles in the transcriptional silencing of transposons.

### The four CLSY members antagonistically regulate DNA methylation

**Fig. 9:**
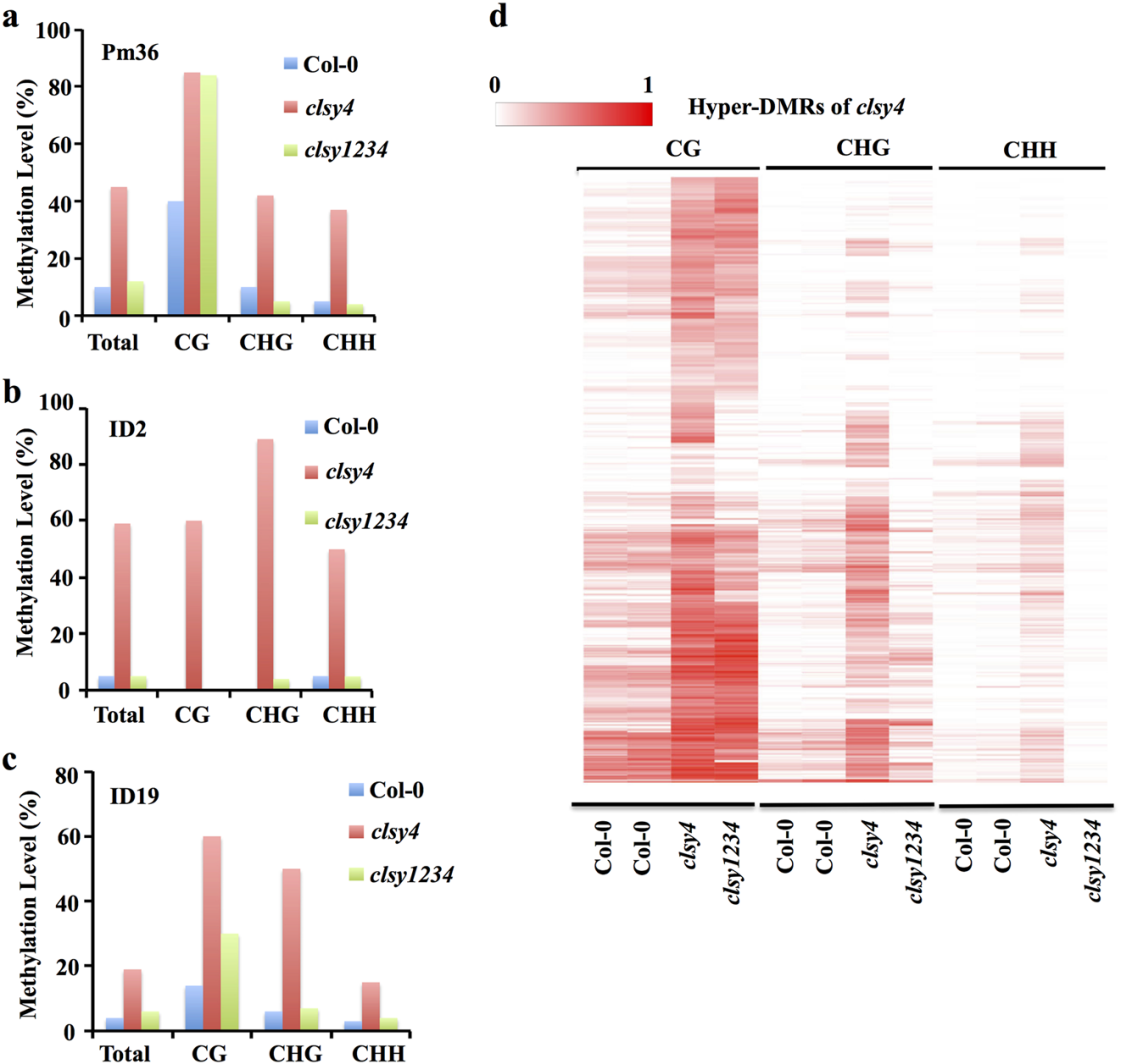
DNA hypermethylation in *clsy4* depends on the other three CLSYs. **a**–**c** DNA methylation analysis by individual locus bisulfite sequencing of the hyper-DMRs Pm36 (**a**), ID2 (**b**), and ID19 (**c**) in the wild type, *clsy4*, and *clsy1234*. **d** Methylation levels of CG, CHG, and CHH at *clsy4* hyper-DMRs

Because the DNA hypermethylation in *clsy4* mutants depends on RdDM, which in turn requires the redundant function of the four CLSY proteins, we suspected that the DNA demethylation function of one CLSY is antagonized by the other CLSY proteins. To examine this possibility, we examined methylation levels of three *clsy4* hyper-DMRs using individual locus bisulfite sequencing. At Pm36, the non-CG hypermethylation of *clsy4* was suppressed in *clsy1234* mutant plants, while the CG hypermethylation did not change in the quadruple mutant (Fig. 9a). At the other two *clsy4* hyper-DMRs, ID2 and ID19, hypermethylation was reduced or abolished in all three contexts in the quadruple mutant (Fig. 9b, c). In addition, Integrated Genome Viewer (IGV) examination of randomly selected *clsy4* hyper-DMRs from whole-genome bisulfite sequencing results also showed a loss of DNA hypermethylation in *clsy1234* compared to *clsy4* (Supplementary Fig. S12a–f). These results revealed that, at some RdDM target loci, CLSY4-dependent DNA demethylation counteracts the DNA methylation mediated by the other three CLSY proteins, demonstrating an important role of the CLSY proteins in regulating DNA methylation dynamics.

On a whole-genome scale, CHG and CHH methylation at *clsy4* hyper-DMRs was significantly reduced in *clsy1234* compared to *clsy4*, as shown by boxplot analysis (Supplementary Fig. S12g–j). Moreover, heatmap analysis showed that the increased CHG and CHH DNA methylation at most *clsy4* hyper-DMRs was lost in *clsy1234*, whereas the CG hypermethylation at *clsy4* hyper-DMRs largely remained in *clsy1234* (Fig. 9d). These genome-wide patterns further supported the conclusion that CLSY4 antagonizes the other CLSYs in balancing DNA methylation levels at specific genomic loci, although CLSY4 also positively contributes to DNA methylation at some other loci in a way that is genetically redundant with the other CLSYs.

We further investigated the relationship among the four CLSYs by comparing all *clsy* single mutants with *clsy1234*. At *clsy1*, *clsy2*, or *clsy3* hyper-DMRs, DNA hypermethylation was largely abolished in *clsy1234*, as shown by heatmap analyses (Supplementary Fig. S13a–c). In addition, at these *clsy* single mutant hyper-DMRs, cytosine methylation levels and especially CHG and CHH methylation levels were reduced in *clsy1234* (Supplementary Fig. S14a–c), indicating a general antagonistic relationship among the four CLSY members in regulating DNA methylation dynamics. Consistent with this inference, the hyper-DMRs in *clsy123*, *clsy134*, and *clsy234* were also largely reduced in the *clsy1234* mutant (Supplementary Fig. S13d–f), with CHG and CHH methylation reduced more than CG methylation (Supplementary Fig. S15a–c).

## Discussion

Arabidopsis CLSY1 was first identified in a genetic screen for cellular factors required for gene silencing induced by double-stranded RNAs and was demonstrated to function with RDR2 and NRPD1 in the production of 24-nt siRNA at endogenous loci^20^. However, DNA methylome analysis of multiple RdDM mutants showed that the methylation level at RdDM target loci was only weakly reduced in *clsy1* mutants^21^, suggesting that other chromatin-remodeling proteins likely work together with CLSY1 in siRNA biogenesis. Later, CLSY1 and its three paralogs, CLSY2, CLSY3, and CLSY4, were identified as Pol IV-associated proteins, suggesting that these proteins probably function redundantly in siRNA production in the RdDM pathway^34^. Here we profiled the DNA methylomes of single, triple, and quadruple *clsy* mutants and found that the DNA methylation of RdDM target loci is indeed cooperatively controlled by the four CLSY proteins. We found that 24-nt siRNAs were almost eliminated in the *clsy1234* mutant. Although the detailed molecular mechanism underlying the involvement of the CLSYs in Pol IV function remains to be elucidated, chromatin enrichment of DTF1/SHH1, the cofactor important for Pol IV recruitment and 24-nt siRNA production at a subset of RdDM loci^18,19^, was reduced in *clsy1234* at several of the tested genomic regions.

In this study, CLSY4 (a close homolog of CLSY1) was originally identified in our genetic screen for DNA demethylation factors. More than 1000 hyper-DMRs were found in *clsy4* mutant plants, indicating that CLSY4 is important for genome-wide DNA demethylation. *ROS1* expression was not reduced in *clsy4* mutants, indicating that CLSY4 does not function indirectly in demethylation by being an RdDM factor that controls *ROS1* expression. The biochemical function of CLSY4 in DNA demethylation is unknown, and we have not observed a physical association between ROS1 and any of the four CLSYs (unpublished data). Nevertheless, we found that the nucleosome occupancy was reduced in *clsy4* at several tested genomic loci targeted by active DNA demethylation. ATP-dependent chromatin-remodeling proteins generally function in nucleosome sliding, eviction, and histone exchange on chromatin^41^. It is possible that active DNA demethylation at some genomic loci may somehow involve CLSY-mediated nucleosome movement. In support of this notion of a chromatin remodeling functioning in active DNA demethylation, Ikeda et al. have documented that the FACT histone chaperone component SSRP1 plays an important role in DME-mediated active DNA demethylation and gene imprinting^42^.

With the use of *clsy4nrpd1* and *clsy1234* mutants, our genetic analysis demonstrated that DNA hypermethylation in *clsy4* depends on the RdDM pathway, a pattern resembling that of the *ros1* mutant. It is possible that, besides CLSY4, the other three CLSYs also directly regulate DNA demethylation at discrete genomic regions, because hundreds of hyper-DMRs were identified in the single and triple *clsy* mutants. Those hyper-DMRs also depended on the RdDM pathway, because DNA hypermethylation was largely abolished in the *clsy1234* mutant, in which DNA methylation and siRNA accumulation at RdDM target loci was eliminated. Therefore, the dual function of CLSY proteins and especially of CLSY4, whose mutation results in the highest number of hyper-DMRs, in DNA methylation via RdDM and in active DNA demethylation mediated by the ROS1 family of DNA demethylases represents a new mechanism underlying the dynamic regulation of DNA methylation. DNA methylation and demethylation activities were previously determined to be coordinated by a “methylstat”, a methylation-sensitive cis-element at the *ROS1* promoter^30,31^. The current results suggest that chromatin remodeling by the four CLSYs is another mechanism for the coordination of DNA methylation and demethylation.

Mutations in the CLSYs led to increased DNA methylation at certain genomic sites but deceased DNA methylation at other sites. The opposite DNA methylation phenotypes in the *clsy* mutants raises the possibility that other epigenetic marks, like histone modifications and pre-existing DNA methylation together with nucleosome occupancy regulated by CLSYs, may collectively determine whether a region undergoes RdDM or active DNA demethylation. The chromatin status regulated by the CLSYs is only one of the factors determining the dominance of RdDM or active DNA demethylation. How RdDM and active DNA demethylation differ in their requirements for chromatin environments warrants additional study.

## Materials and methods

### Plant materials and growth conditions

All Arabidopsis plants used in this study were in the Col-0 background. The *clsy1-3* mutant was reported previously^20^. All other mutants (Supplementary Table S3) were either ordered from the Arabidopsis Biological Resource Center (http://www.arabidopsis.org) or made in this study. The oligos used for genotyping and making higher order mutants are listed in Supplementary Table S4. Plants that were subjected to DNA methylation and small RNA analyses were 2-week-old seedlings grown on 1/2 strength Murashige Skoog (MS) medium supplemented with 0.7% agar and 1% sucrose. The seedlings were grown in growth chambers (Percival, Inc.) at 22 °C with 16 h of light and 8 h of darkness.

### Chop-PCR and genetic screen

The T-DNA mutants (Supplementary Table S1) were grown in soil for 5 weeks. Four plants of each genotype were selected for DNA extraction by the CTAB method. About 2 µg of DNA of each plant was diluted and digested with HhaI or HpaII in a 20-µl reaction mixture for 8 h. The PCR reaction used 1 µl of enzyme-digested DNA as the template. Agarose (1%) gels were used to compare the amplified DNA signal, with the wild type and *ros1-4* as controls. Candidate mutants were then further genotyped and confirmed.

### Individual locus bisulfite sequencing

After the genomic DNA was isolated from 4-week-old Arabidopsis plants using the Plant DNeasy Mini Kit (Qiagen), about 100 ng of genomic DNA was subjected to sodium bisulfite treatment and purification using the BisulFlash DNA Modification Kit (Epigentek) and following the manufacturer’s protocol. A 1 µl volume of bisulfite-converted DNA was used for PCR reaction with oligos designed for specific target regions (Supplementary Table S4). The PCR products were ligated into the pMD18-T vector (Takara, Japan). The ligation product was transformed into DH5α competent cells, and at least 15 single clones were sequenced for each type of transformant. The sequencing results were aligned and analyzed using CyMate (http://www.cymate.org).

### Whole-genome bisulfite sequencing and data analysis

Genomic DNA was extracted from 2-week-old seedlings grown in 1/2 strength MS medium and was sent to the Beijing Genomics Institute (Shenzhen, China) for bisulfite treatment, library construction, and high-throughput sequencing. Clean reads were generated by trimming adaptor and low-quality sequences (q < 20) and then were mapped to the Arabidopsis genome (TAIR 10) using BSMAP (Bisulfite Sequence Mapping Program) and allowing two mismatches. Differentially methylated regions (DMRs) were identified as previously described^16^. First, the cytosines with a read coverage < 5 were filtered out. The DNA methylation level in every 200-bp window with a step size of 50 bp was compared between control and mutant plants using Fisher’s exact test. *P*-values calculated from the tests were then adjusted using the Benjamini-Hochberg method to control for the false discovery rate (FDR). Windows with an adjusted *P*-value < 0.05 were then tested for the number of DMCs (differentially methylated cytosines), which were defined as cytosines with a *P*-value < 0.01 in Fisher’s exact test. The DMR-identified windows with at least 7 DMCs and ≥ 1.5-fold change in DNA methylation levels were combined to generate the final list of DMRs if the gap length between two windows was ≤ 100 bp.

All of the figures related to DNA methylomes were generated using R (https://www.r-project.org/). The heatmaps were generated using “heatmap.2” from “gplots” library, with the following parameters: Colv = FALSE, dendrogram = “row”, trace = “none”, and labRow = “NA”. The methylation data were not normalized for generating the heatmaps.

### Genome-wide small RNA sequencing and analysis

Total RNA was isolated from 2-week-old seedlings with TRIzole reagent (Sigma) and the standard protocol. Total RNA was separated on PAGE gels and < 35-nt fractions were cut and purified for small RNA library preparation and sequencing at BGI (Shenzhen, China). After sequencing, adapter sequences were trimmed, and clean reads from 18 to 30 nt were mapped to the Arabidopsis genome (TAIR10) using Bowtie with parameter “-v 0 –k 10”^16^ Reads were mapped to annotated structural RNAs including tRNAs, rRNAs, snRNAs, and snoRNAs were excluded. Read counts were normalized to Reads Per Ten Million (RPTM) based on total mapped reads. The “hits-normalized abundance” (HNA) values were calculated by dividing the normalized abundance (in RPTM) for each small RNA hit, where a hit was simply defined as the number of loci at which a given sequence perfectly matched the genome^16^, and the values were used for boxplot generation.

### Chromatin immunoprecipitation assay

The chromatin immunoprecipitation was conducted using 2-week-old seedlings of the wild type Col-0, DTF1-MYC/*clsy1234*, and DTF1-MYC/Col-0 grown in 1/2 strength MS solid medium as previously described^43^. The antibody used for DTF1 ChIP assays was anti-MYC (16–219, Millipore). The DNA was diluted in 50 μl of TE, and a 1.5 μl volume was used for each qPCR reaction. The relative enrichment to input was calculated from three biological replicates.

Nuclei were extracted from 2-week-old Arabidopsis seedlings grown in 1/2 strength MS medium as previously described^38^ and were digested with Micrococcal Nuclease (MNase; NEB) before chromatin immunoprecipitation was conducted using antibodies against H3 (Abcam, ab1791) as previously reported^44^.

### Small RNA extraction and northern blotting

After total RNA was extracted from 2-week-old Arabidopsis seedlings with TRIzole reagent (Sigma) and the standard protocol, the small RNA fraction was precipitated by the PEG method. In brief, equal volumes of a PEG8000 solution (20% PEG 8000, 1 M NaCl) were added to the total RNA. After centrifugation at 16000 *g* at 4 °C, an equal volume of isoproponal and 0.1 volume of 3 M NaAC were added to the supernatant. Following another centrifugation at 16000 *g* at 4 °C, the small RNA pellet was re-suspended in DEPC-treated water. For each sample, small RNAs from about 100 µg of total RNA were separated on a 17% polyacrylamide gel, which was electrotransferred to a Hybond N+ membrane (GE Lifesciences). Membranes were cross-linked, incubated for 2 h at 80 °C, and hybridized overnight at 38 °C with ^32^P-labelled DNA probes or oligonucleotides (listed in Supplementary Table S4) in PerfectHyb buffer (Sigma). The washed membranes were then exposed in the presence of X-ray film at −80 °C for 7 days.

### Data access

The sequencing data generated in this study have been deposited in the NCBI Gene Expression Omnibus (GEO; http://www.ncbi.nlm. nih.gov/geo/) and are accessible through accession number GSE110338.

## Electronic supplementary material

Supplementary Information

## Electronic supplementary material

**Supplementary Information** accompanies the paper at (10.1038/s41421-018-0056-8).
