# Supplementary material for: Four putative SWI2/SNF2 chromatin remodelers have dual roles in regulating DNA methylation in Arabidopsis
Source: Cell Discov. 2018 Oct 16;4:55. doi: 10.1038/s41421-018-0056-8 (PMC6189096; doi:10.1038/s41421-018-0056-8)
Supplement: Supplementary file 1 — Supplementary Information [file 41421_2018_56_MOESM1_ESM.pdf]

Supplementary Figure 1

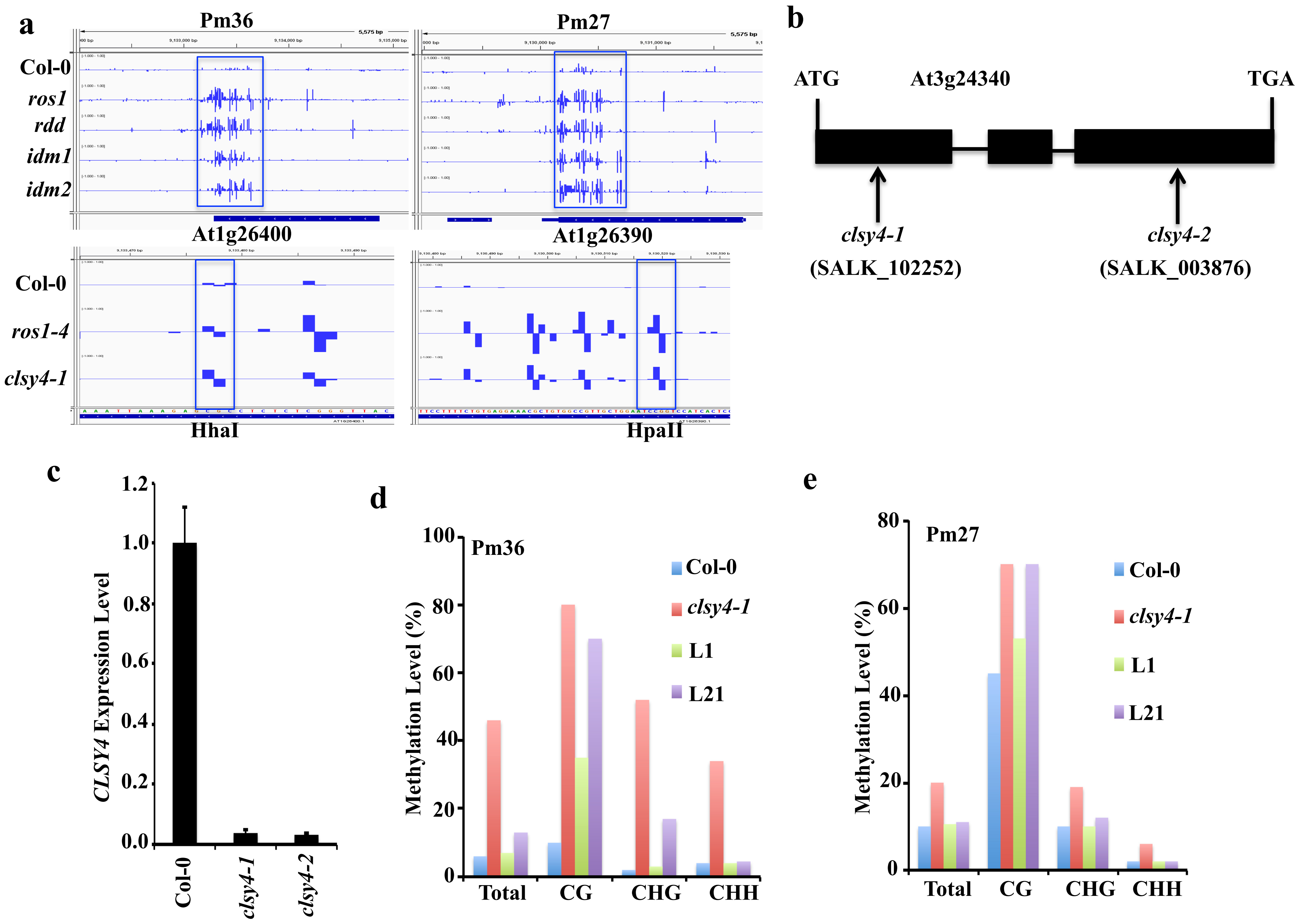

Supplementary Figure 2

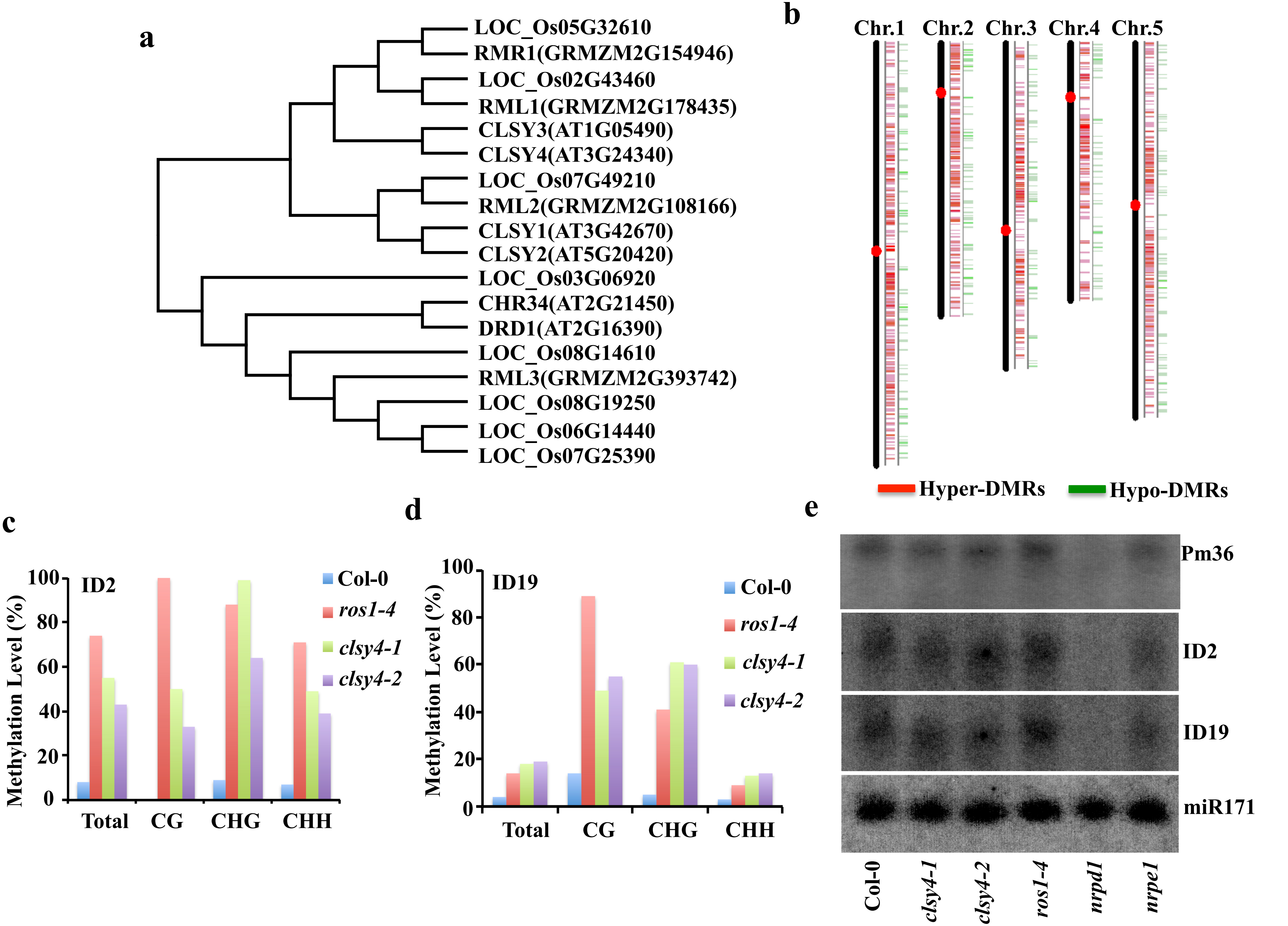

Supplementary Figure 3

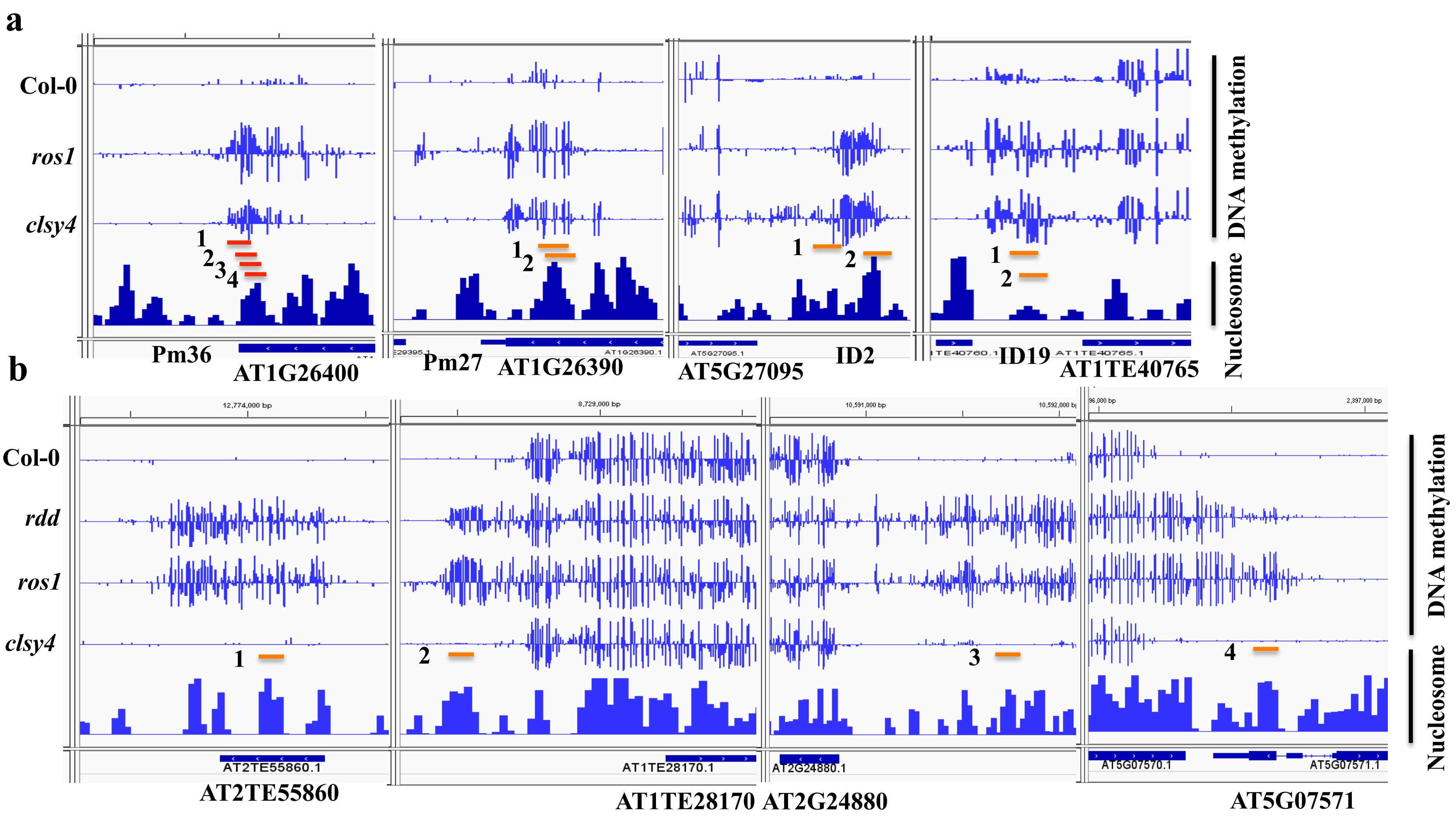

Supplementary Figure 4

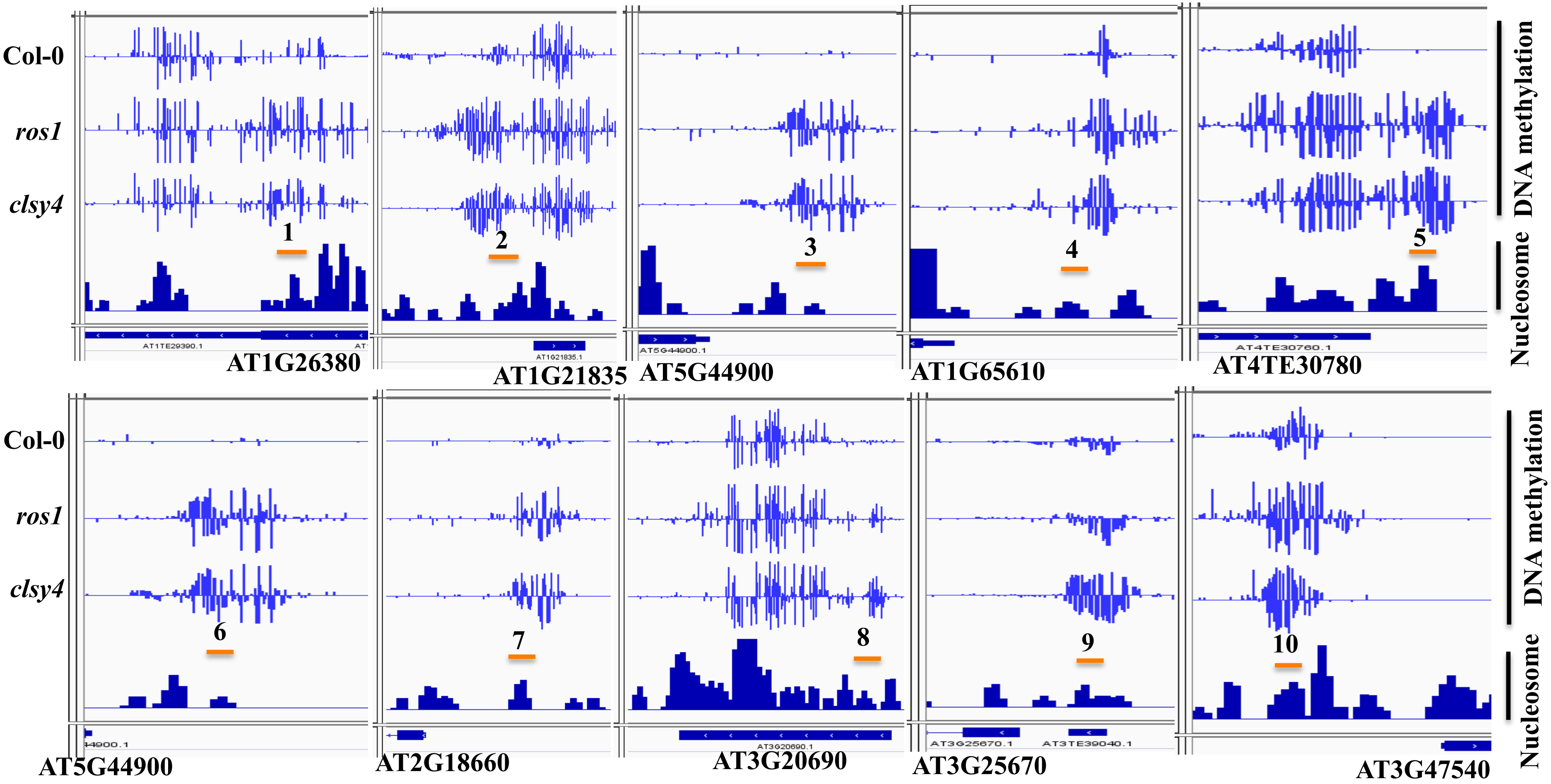

Supplementary Figure 5

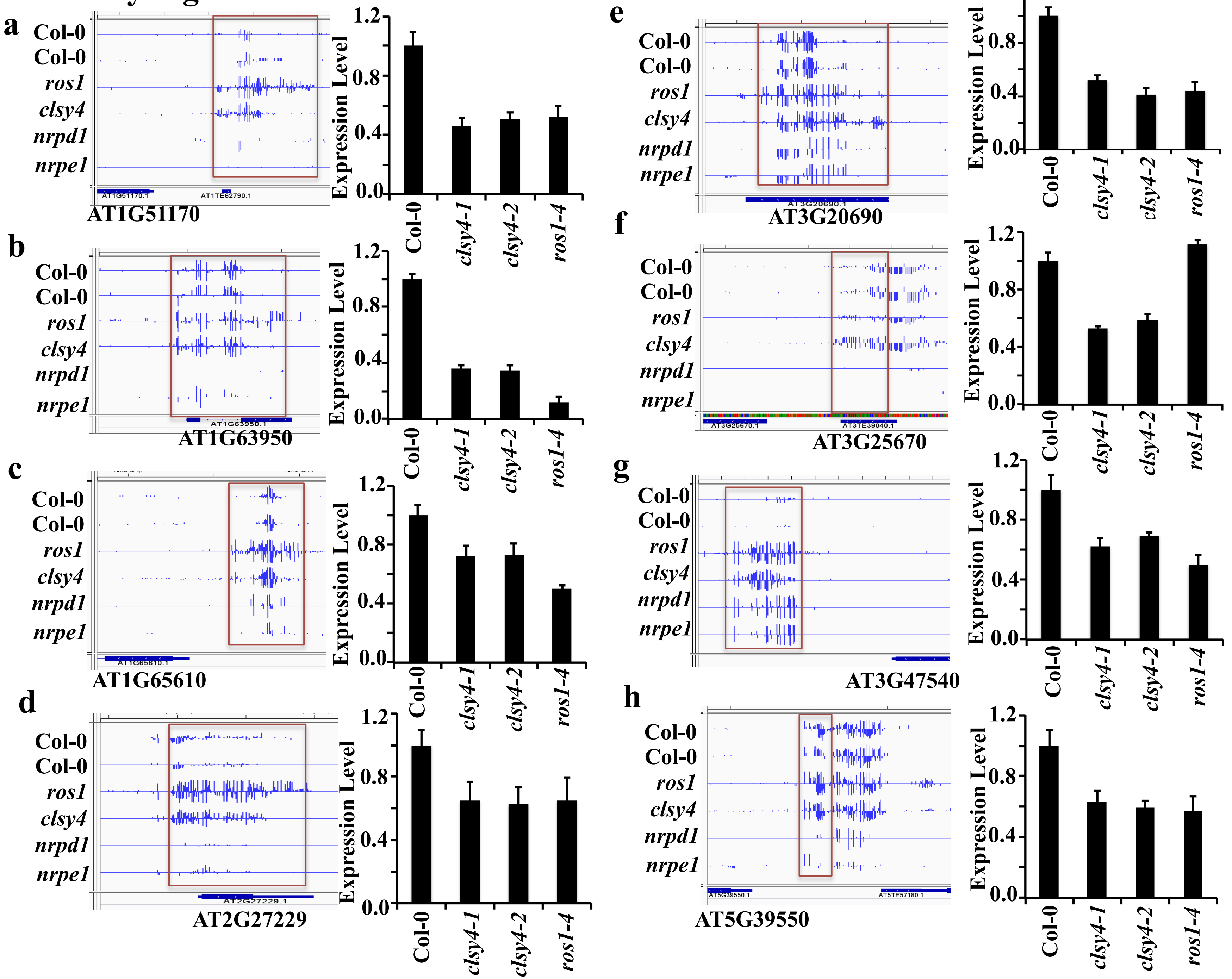

Supplementary Figure 6

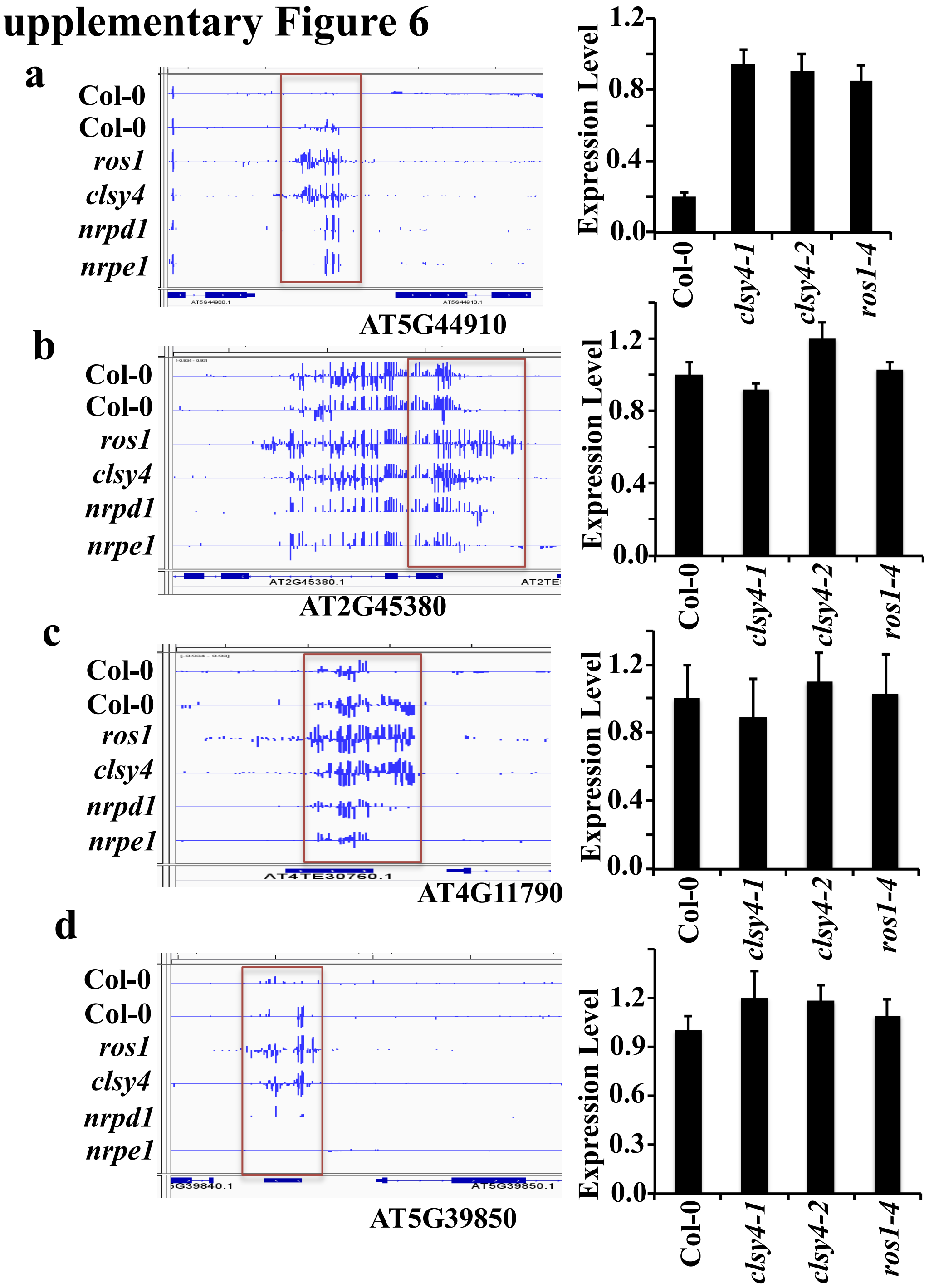

Supplementary Figure 7

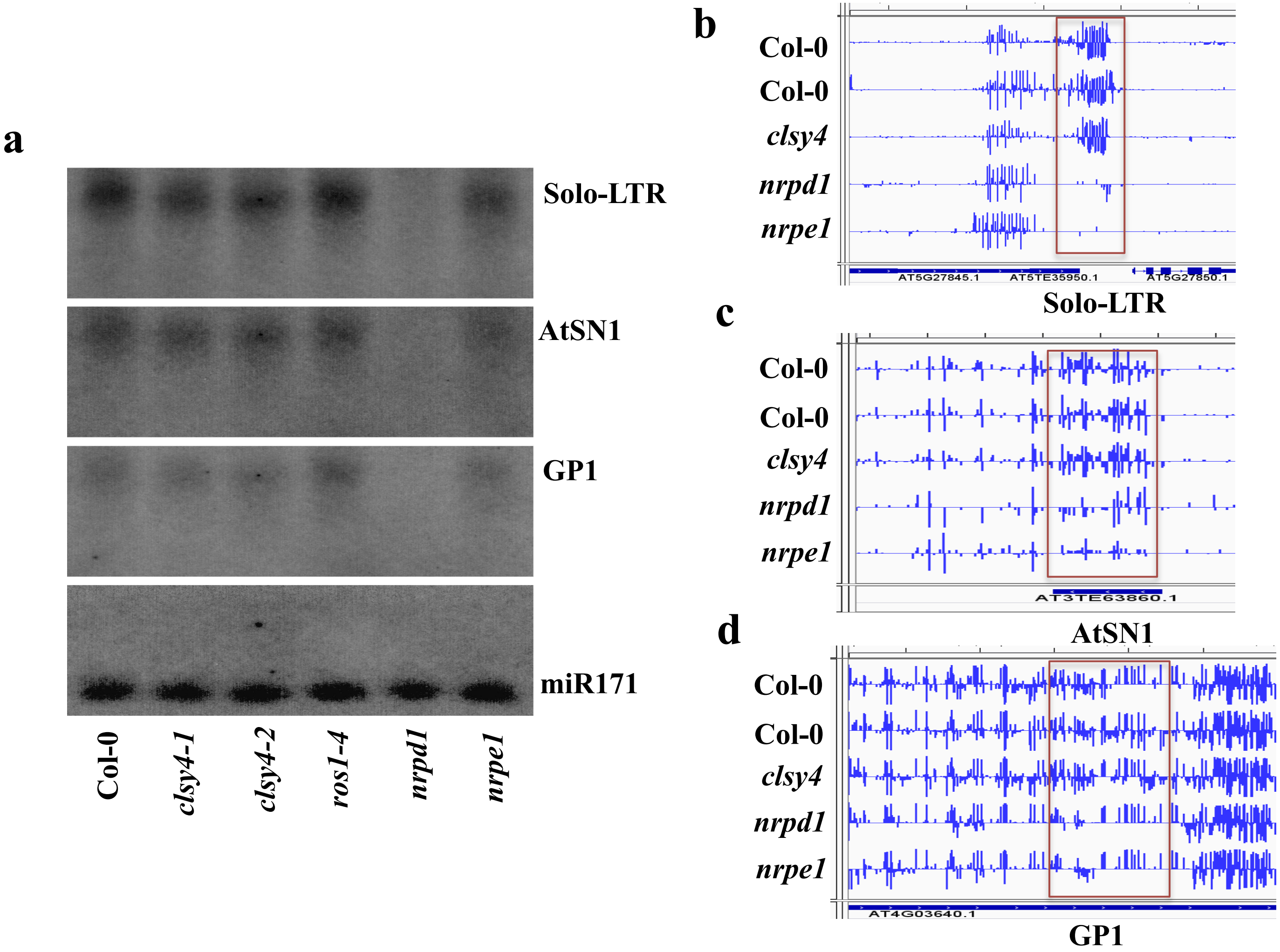

Supplementary Figure 8

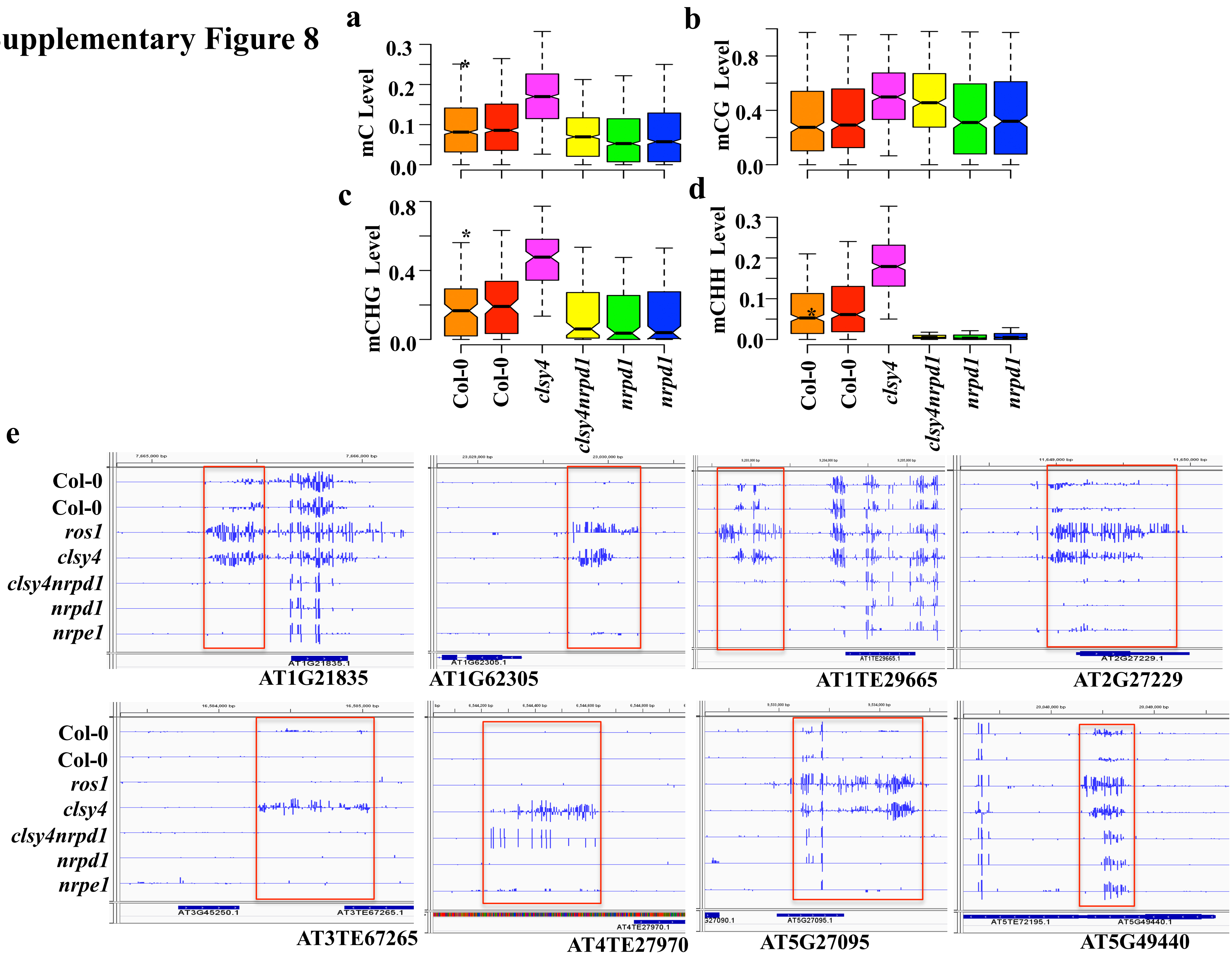

Supplementary Figure 9

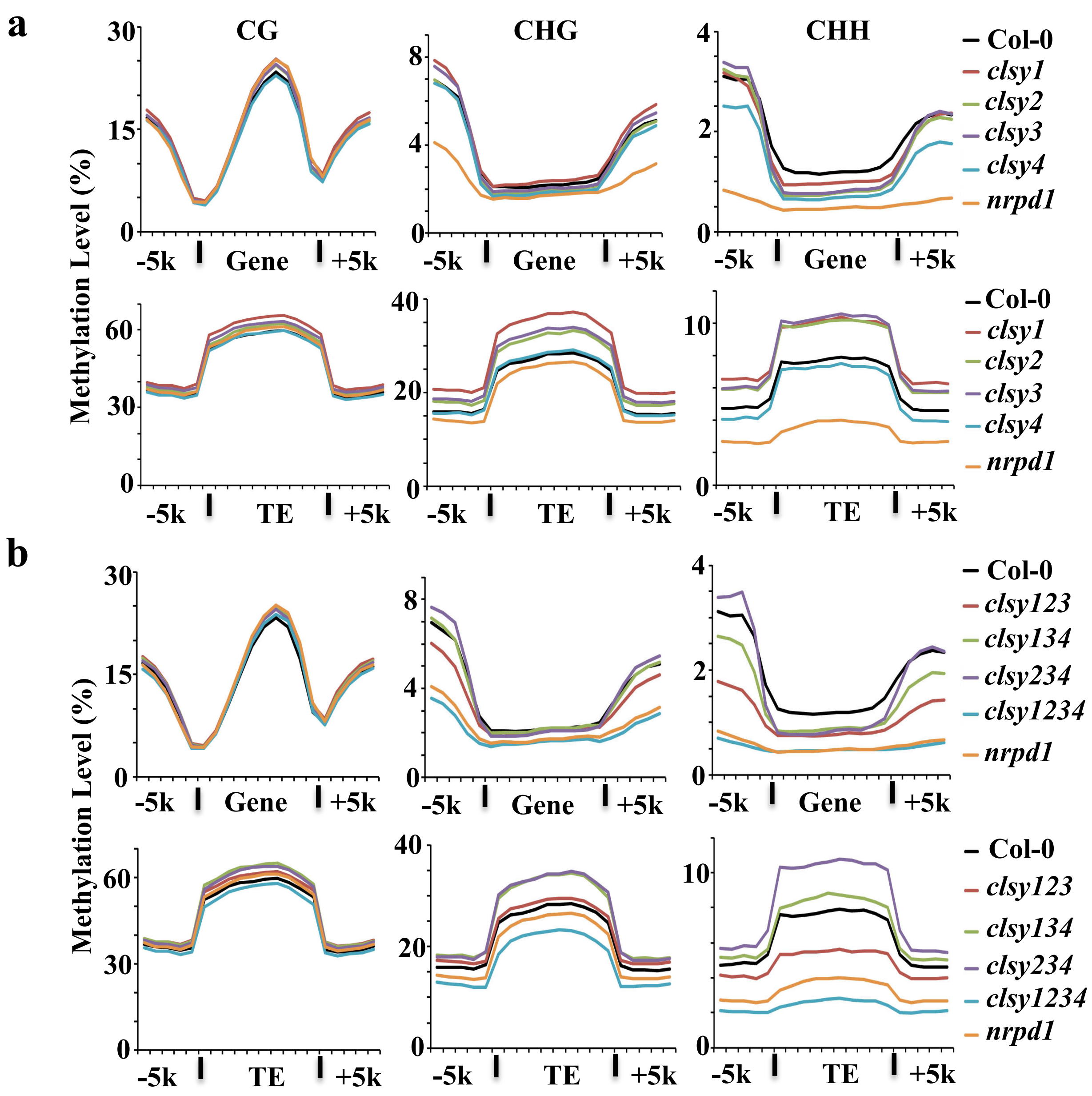

Supplementary Figure 10

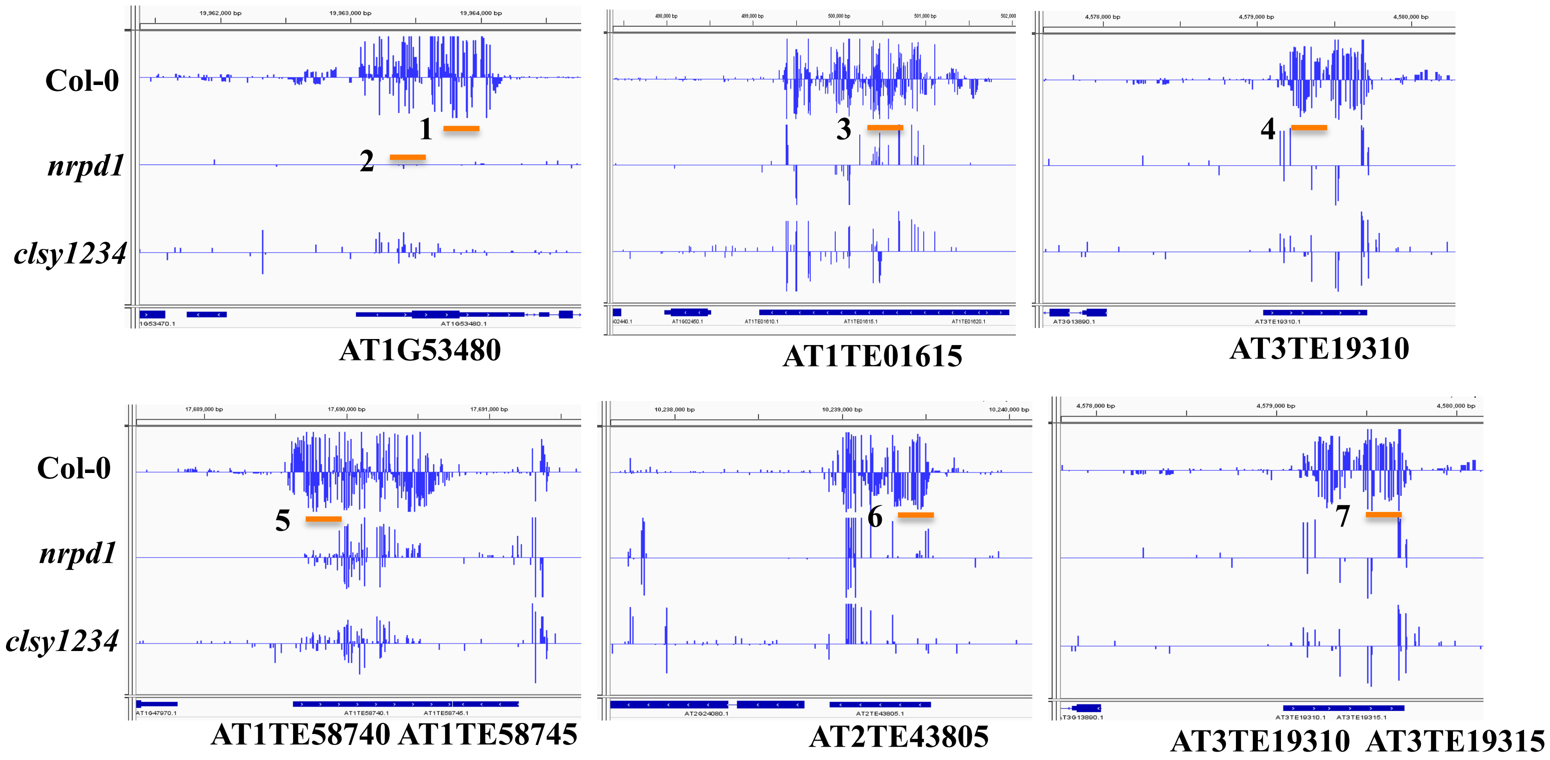

Supplementary Figure 11

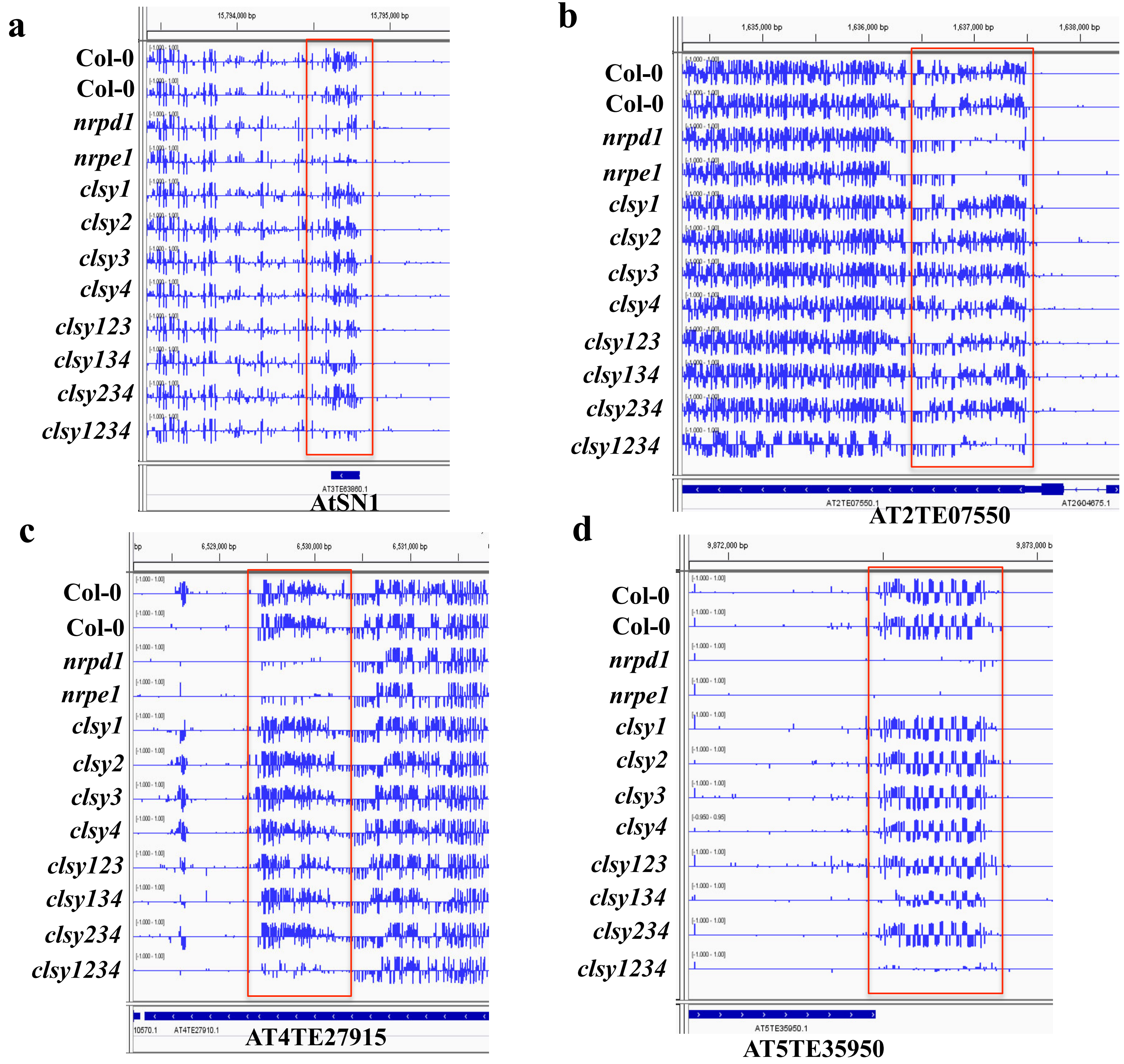

Supplementary Figure 12

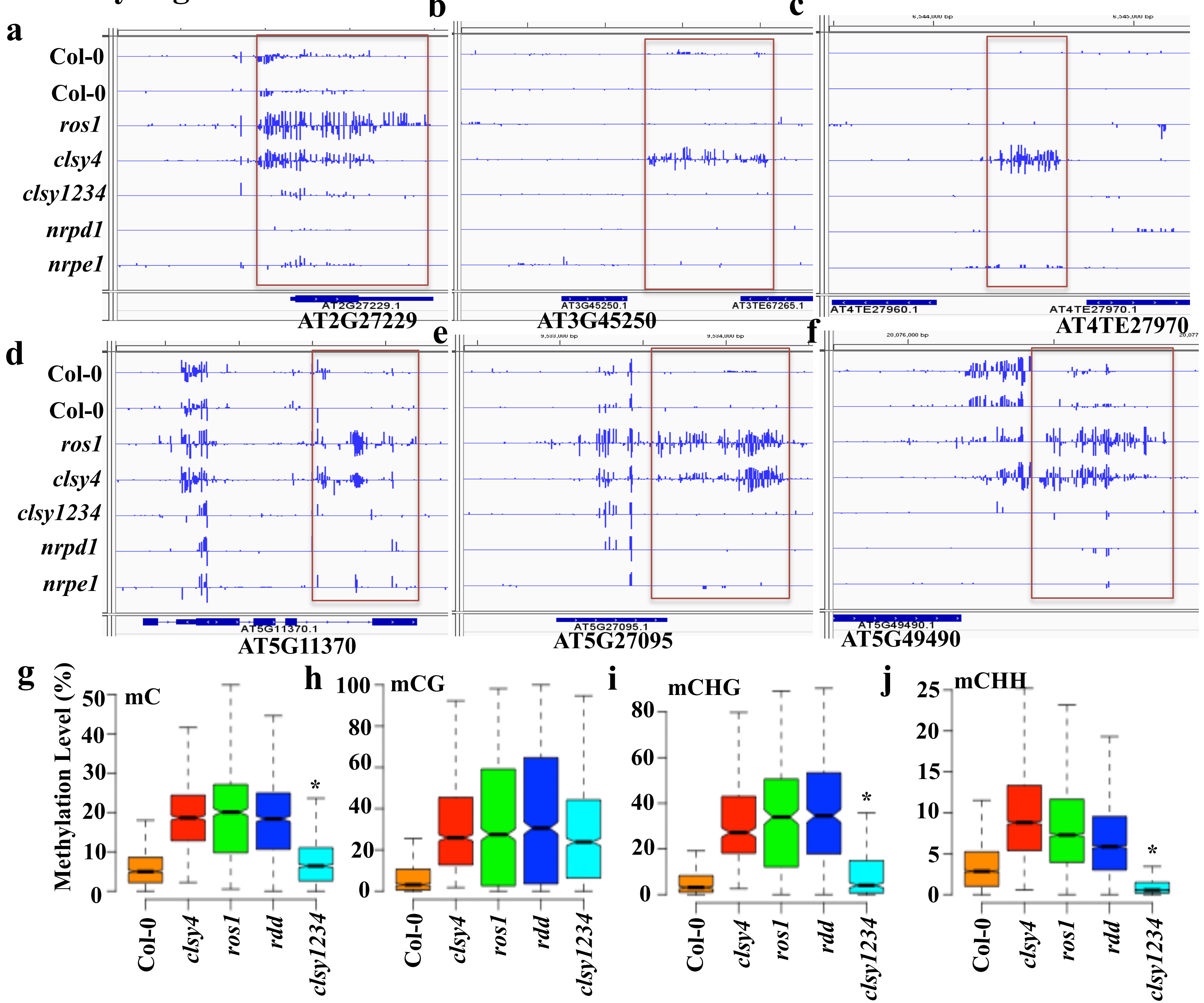

Supplementary Figure 13

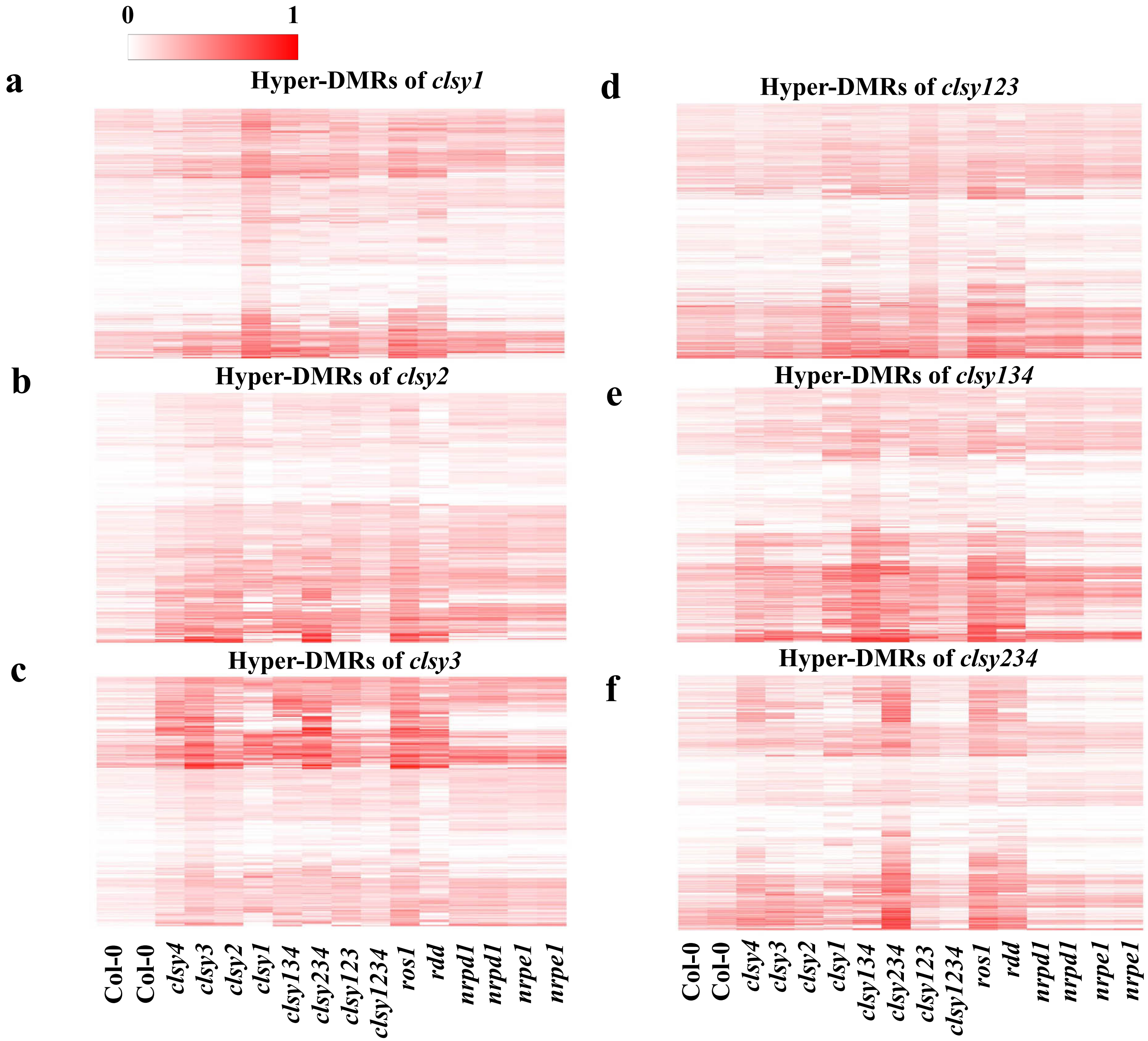

Supplementary Figure 14

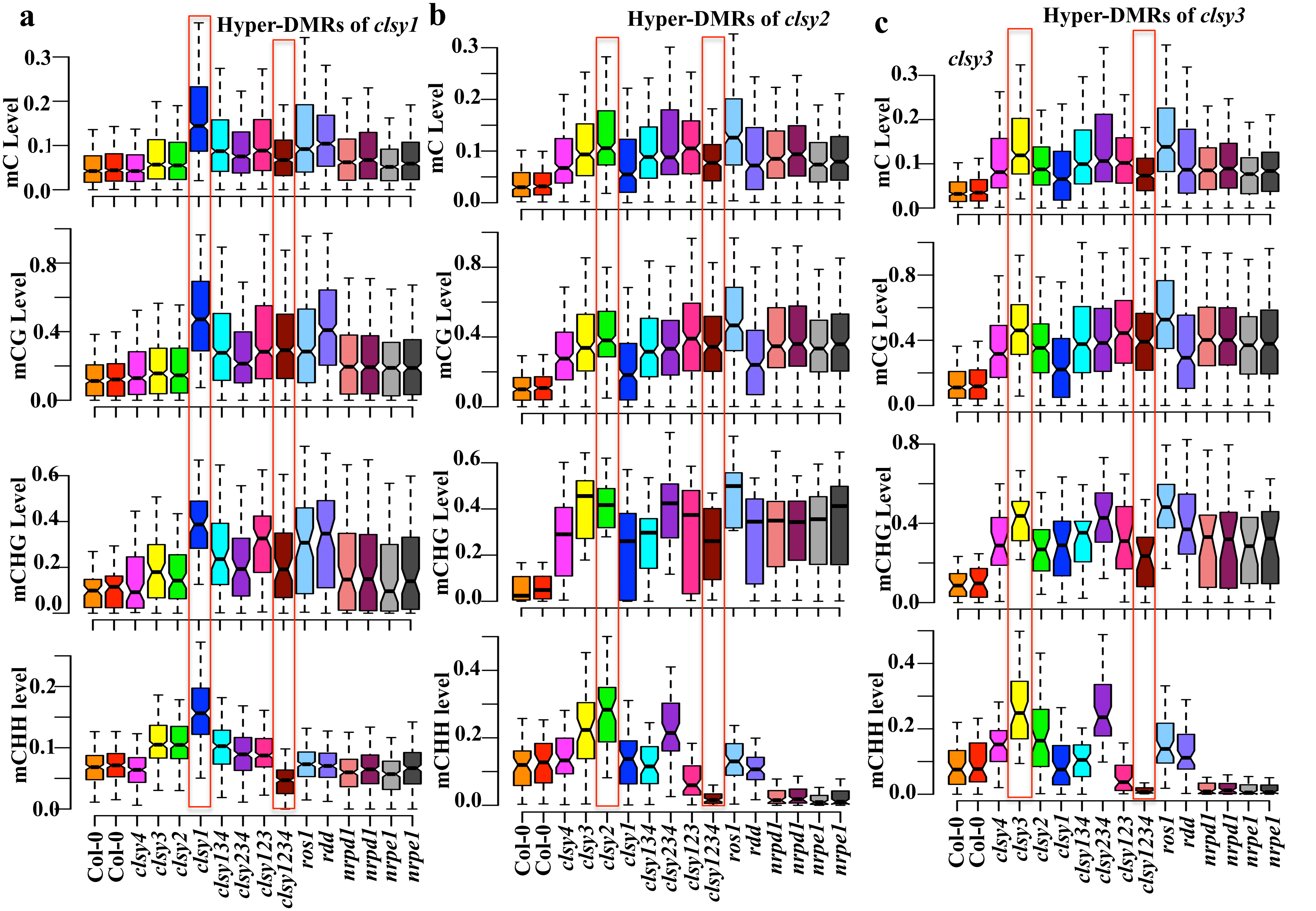

Supplementary Figure 15

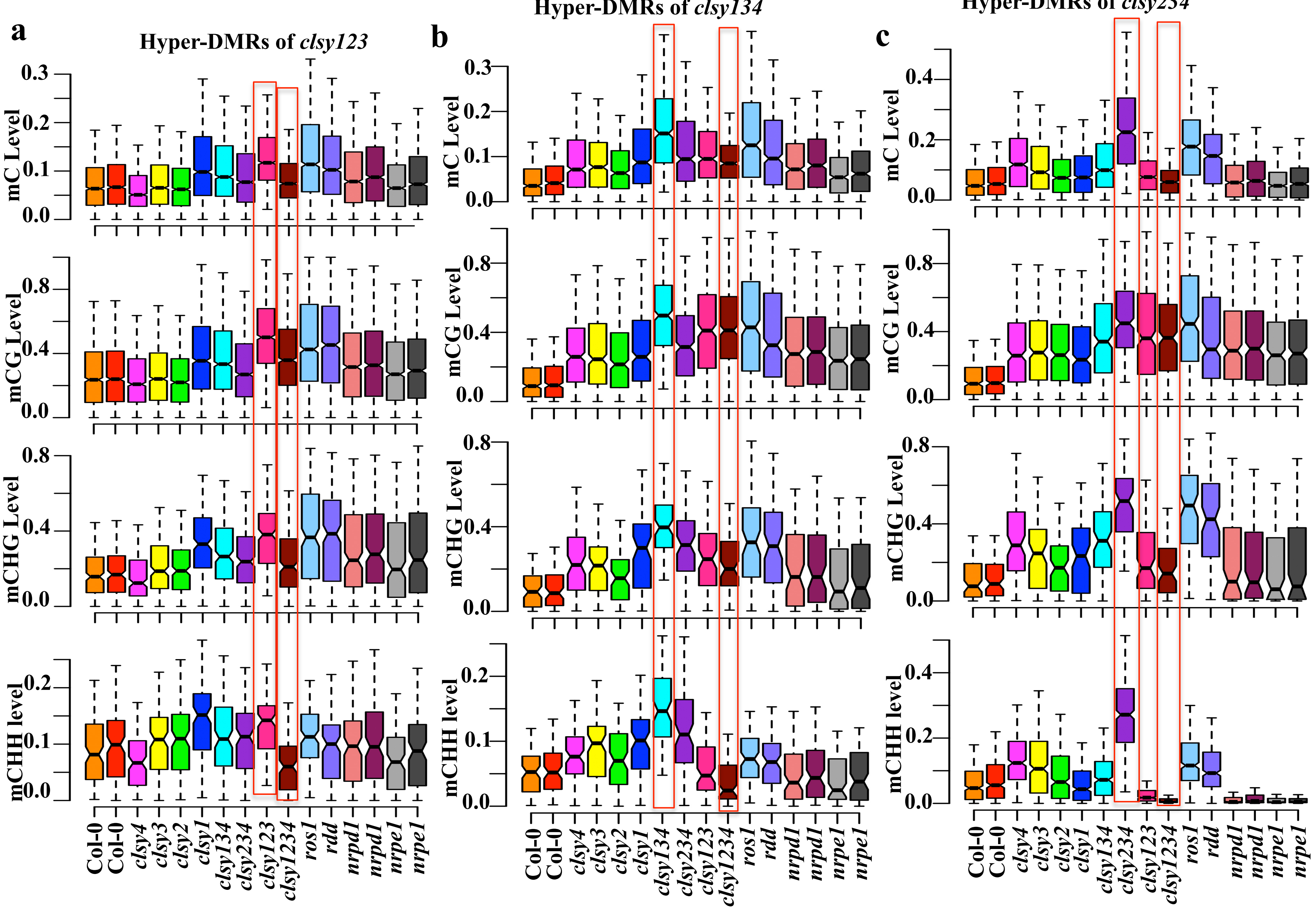

Supplementary Figure 1. The mutation in *CLSY4* was responsible for DNA hypermethylation.

(a) Integrated Genome Viewer display of the genomic location and DNA methylation level of the chop-PCR markers Pm36 and Pm27 in the indicated genotypes. The cytosine methylation levels at enzyme cutting sites are indicated by the IGV screenshots. (b) Schematic diagram showing the positions of two T-DNA insertions. (c) The *CLSY4* transcript levels in *clsy4* mutants as indicated by real-time PCR assay. (d) DNA methylation analysis of Pm36 in *clsy4* and complementation lines. (e) DNA methylation analysis of Pm27 in *clsy4* and complementation lines.

Supplementary Figure 2. There was no increase in siRNA at *clsy4* hyper-DMRs.

(a) The phylogenetic tree of plant-specific SNF2 proteins from Arabidopsis, rice, and maize generated by MEGA 5.2.2. (b) The distribution of DMRs of *clsy4* in the genome, with the red circle representing the centromere. (c) Individual locus bisulfite sequencing at a *clsy4* hyper-DMR, ID2. (d) Individual locus bisulfite sequencing at a *clsy4* hyper-DMR, ID19. (e) siRNA abundance at Pm36, ID2, and ID19 in *clsy4* and RdDM mutants measured by northern blotting.

Supplementary Figure 3. DNA methylation levels and nucleosome positions of the regions tested in ChIP assays for nucleosome positioning.

(a) Integrated Genome Viewer (IGV) display of the DNA methylation level in Col-0, *ros1-4*, and *clsy4-1*, and nucleosome position in Col-0[37]. The regions for ChIP-PCR are indicated by the orange lines. The identities of the common DMRs of *clsy4* and *rdd* are shown below each IGV snapshot. (b) Integrated Genome Viewer (IGV) display of the DNA methylation levels in Col-0, *rdd*, *ros1-4*, and *clsy4-1*, and nucleosome positioning in Col-0 at four *rdd*-specific hyper-DMRs.

Supplementary Figure 4. DNA methylation levels and nucleosome positioning of 10 hyper-DMRs of *clsy4*.

Integrated Genome Viewer (IGV) display of the DNA methylation levels in Col-0, *ros1-4*, and *clsy4-1*, and nucleosome positioning in Col-0[37]. The regions for ChIP-PCR are indicated by the orange lines.

Supplementary Figure 5. Hyper-DMRs in the *clsy4* mutant repress nearby gene expression.

(a-h) The IGV images on the left show the DNA methylation levels at hyper-DMRs in various genotypes, and IGV images on the right show the relative expression levels of nearby genes in the wild type *clsy4*, and *ros1*. Bars indicate the standard errors of three biological replicates.

Supplementary Figure 6. Effect of hyper-DMRs on nearby gene expression.

(a) A hyper-DMR in the *clsy4* mutant increases nearby gene expression. (b-d) Hyper-DMRs in the *clsy4* mutant have no substantial effect on nearby gene expression. Bars indicate the standard errors of three biological replicates.

Supplementary Figure 7. The *clsy4* mutation does not affect siRNA abundance or DNA methylation level of several known RdDM loci.

(a) Northern blotting showing the abundance of 24-nt siRNA at Solo-LTR, AtSN1, and GP1 (three RdDM target loci in the indicated genotypes). miR171 served as a loading control. (b-d) Snapshots showing the DNA methylation levels of Solo-LTR, AtSN1, and GP1 in *clsy4, nrpd1*, and *nrpe1* mutants.

Supplementary Figure 8. DNA hypermethylation in *clsy4* depends on RdDM.

(a-d) DNA methylation levels of C, CG, CHG, and CHH at *clsy4* hyper-DMRs in the indicated genotypes. * indicates significant difference between *clsy4* and *clsy4nrpd1* (P <0.01, Wilcoxon sum test). (e) Snapshots showing the DNA methylation of eight hyper-DMRs of *clsy4* in various genotypes.

Supplementary Figure 9. Role of the CLSYs in regulating DNA methylation at genes and transposons.

Methylation levels of CG, CHG, and CHH at genes and transposons and their flanking regions in (a) the *clsy* single mutant and in (b) the *clsy* triple and quadruple mutants. The wild type and *nrpd1* served as controls.

Supplementary Figure 10. DNA methylation levels of hypo-DMRs of *clsy1234*.

Integrated Genome Viewer (IGV) display of the DNA methylation levels in Col-0, *nrpd1*, and *clsy1234*. The regions for ChIP-PCR are indicated by the orange line.

Supplementary Figure 11. The four CLSYs redundantly regulate DNA methylation at transposons.

Snapshots showing the DNA methylation levels at RdDM-targeted transposons AtSN1 (a), AT2TE07550 (b), AT4TE27915 (c), and AT2TE07550 (d) in various *clsy* mutants.

Supplementary Figure 12. DNA hypermethylation in *clsy4* depends on the other three CLSYs.

(a-f) Snapshots showing the DNA methylation level of *clsy4* hyper-DMRs in the indicated genotypes. (g-j) Boxplots showing the DNA methylation of C, CG, CHG, and CHH at *clsy4* hyper-DMRs in the indicated genotypes. * indicates significant difference between *clsy4* and *clsy1234* ($P < 0.01$, Wilcoxon sum test).

Supplementary Figure 13. The four CLSYs antagonistically regulate DNA methylation and demethylation at the hyper-DMRs in individual *clsy* mutants.

Total C methylation levels of various mutants at (a) *clsy1* hyper-DMRs, (b) *clsy2* hyper-DMRs, (c) *clsy3* hyper-DMRs, (d) *clsy123* hyper-DMRs, (e) *clsy134* hyper-DMRs, and (f) *clsy234* hyper-DMRs.

Supplementary Figure 14. DNA hypermethylation of *clsy* single mutants depends on CLSYs.

Boxplot showing the DNA methylation level of C, CG, CHG, and CHH at hyper-DMRs of (a) *clsy1*, (b) *clsy2*, and (c) *clsy3* in the indicated genotypes.

Supplemental Figure 15. DNA hypermethylation of *clsy* triple mutants depends on CLSYs.

Boxplot showing the DNA methylation level of C, CG, CHG, and CHH at hyper-DMRs of (a) *clsy123*, (b) *clsy134*, and (c) *clsy234* in the indicated genotypes.

Supplementary Table 1. T-DNA mutants for genetic screen		
**No.**	**Gene ID**	**T-DNA Number**
1	AT5G04290	SALK_001254
2	AT4G32700	SALK_001669
3	AT5G44750	SALK_005721
4	AT1G14790	SALK_007638
5	AT5G45720	SALK_007734
6	AT3G18090	SALK_008535
7	AT2G34710	SALK_008924
8	AT4G19550	SALK_008553
9	AT2G25170	SALK_010693
10	AT2G01280	SALK_011937
11	AT4G24660	SALK_017963
12	AT4G29090	SALK_023866
13	AT5G13240	SALK_026637
14	AT1G08540	SALK_045706
15	AT2G02090	SALK_054130
16	AT4G17460	SALK_059835
17	AT5G20420	SALK_061962
18	AT2G04630	SALK_064868
19	AT1G10520	SALK_075391
20	AT5G13730	SALK_078760
21	AT1G03280	SALK_081307
22	AT3G28920	SALK_085482
23	AT2G19920	SALK_088175
24	AT1G60620	SALK_088247
25	AT4G24790	SALK_092209
26	AT5G42780	SALK_092897
27	AT3G24340	SALK_102252
28	AT5G09380	SALK_113934
29	AT4G31900	SALK_115303
30	AT4G25180	SALK_125873
31	AT4G08350	SALK_126891
32	AT5G18620	SALK_128524
33	AT1G08060	SALK_131757
34	AT1G63160	SALK_127244
35	AT5G22010	SALK_140231
36	AT1G13870	SALK_140551
37	AT3G54490	SALK_148046
38	AT3G19670	SALK_151254
39	AT4G32040	SALK_000339
40	AT5G63950	SALK_007071

41	AT5G65410	SALK_014023
42	AT3G60390	SALK_014055
43	AT4G31900	SALK_014672
44	AT4G21710	SALK_015933
45	AT2G46020	SALK_030046
46	AT1G79840	SALK_039825
47	AT1G67120	SALK_057010
48	AT5G65310	SALK_058055
49	AT2G21450	SALK_059100
50	AT2G34320	SALK_059700
51	AT1G67630	SALK_064094
52	AT1G63210	SALK_086163
53	AT5G44800	SALK_089483
54	AT1G14790	SALK_112300
55	AT1G63020	SALK_143437
56	AT5G04290	SALK_001254
57	AT3G18520	SALK_004027
58	AT1G79350	SALK_004087
59	AT5G44750	SALK_005721
60	AT5G25060	SALK_011832
61	AT3G06290	SALK_013326
62	AT1G63490	SALK_014109
63	AT4G21710	SALK_015933
64	AT5G26610	SALK_018458
65	AT2G13370	SALK_020296
66	AT5G45190	SALK_021004
67	AT5G24740	SALK_021356
68	AT1G34360	SALK_022422
69	AT3G45830	SALK_024862
70	AT4G31880	SALK_024683
71	AT4G39450	SALK_026025
72	AT2G17410	SALK_026835
73	AT1G02080	SALK_030822
74	AT5G12400	SALK_031040
75	AT4G32620	SALK_039205
76	AT3G01460	SALK_039302
77	AT1G09730	SALK_040576
78	AT1G79090	SALK_040660
79	AT4G29790	SALK_042026
80	AT2G44900	SALK_043325
81	AT1G55040	SALK_043472
82	AT5G46190	SALK_051182

83	AT1G76320	SALK_045754
84	AT1G26830	SALK_050756
85	AT1G51520	SALK_053222
86	AT2G19390	SALK_055660
87	AT3G17740	SALK_055926
88	AT3G22170	SALK_059264
89	AT2G27100	SALK_059424
90	AT3G06940	SALK_060069
91	AT3G56850	SALK_061079
92	AT5G58470	SALK_061974
93	AT5G13590	SALK_062434
94	AT3G26400	SALK_064531
95	AT4G36960	SALK_068296
96	AT1G79730	SALK_070632
97	AT5G04240	SALK_074694
98	AT3G10650	SALK_076476
99	AT4G31430	SALK_076754
100	AT5G08220	SALK_077881
101	AT1G80010	SALK_077996
102	AT3G62240	SALK_079716
103	AT2G30470	SALK_088606
104	AT1G13190	SALK_090357
105	AT1G50620	SALK_094181
106	AT1G28420	SALK_099250
107	AT2G47960	SALK_100372
108	AT5G06120	SALK_100541
109	AT1G23860	SALK_100950
110	AT3G09880	SALK_103167
111	AT1G18950	SALK_104865
112	AT3G09360	SALK_108656
113	AT3G04560	SALK_110145
114	AT5G10940	SALK_113565
115	AT5G11530	SALK_114383
116	AT3G07660	SALK_116148
117	AT1G15940	SALK_116162
118	AT2G30800	SALK_117852
119	AT5G09880	SALK_121246
120	AT1G55970	SALK_121407
121	AT2G44950	SALK_122512
122	AT1G80810	SALK_133849
123	AT5G42950	SALK_135013
124	AT5G57360	SALK_135207

125	AT1G48620	SALK_141611
126	AT1G03060	SALK_148574
127	AT3G19670	SALK_151254
128	AT3G20540	SALK_134274
129	AT1G73805	SALK_138476
130	AT1G22130	SALK_066443
131	AT3G23780	SALK_109513
132	AT3G47830	SALK_119297
133	AT5G14920	SALK_054982
134	AT2G38560	SALK_027259
135	AT2G15400	SALK_099705
136	AT5G19910	SALK_035522
137	AT3G13240	SALK_037693
138	AT4G16780	SALK_106790
139	AT2G40030	SALK_029919
140	AT4G30860	SALK_128444
141	AT2G02520	SALK_059227
142	AT2G28290	SALK_110320
143	AT5G48850	SALK_099766
144	AT3G44570	SALK_043100
145	AT1G67500	SALK_067237
146	AT1G64860	SALK_147985
147	AT5G09790	SALK_130607
148	AT2G41340	SALK_134107
149	AT3G07340	SALK_091124
150	AT3G11220	SALK_079193
151	AT5G03740	SALK_129799
152	AT2G24120	SALK_093884
153	AT5G42765	SALK_149886
154	AT3G10010	SALK_131712
155	AT1G49980	SALK_081715
156	AT5G46920	SALK_064659
157	AT4G02020	SALK_109121
158	AT1G63160	SALK_042804
159	AT3G15080	SALK_024707
160	AT2G36610	SALK_083053
161	AT4G21010	SALK_151548
162	AT1G54250	SALK_151800
163	AT2G27120	SALK_025607
164	AT2G44980	SALK_095969
165	AT2G33690	SALK_018685
166	AT3G53920	SALK_009166

167	AT2G31450	SALK_054181
168	AT5G40310	SALK_068417
169	AT1G05900	SALK_013055
170	AT3G09360	SALK_071454
171	AT4G18820	SALK_102798
172	AT2G32160	SALK_118137
173	AT2G19910	SALK_036925
174	AT1G30590	SALK_146255
175	AT4G22390	SALK_136090
176	AT5G25220	SALK_136464
177	AT5G26040	SALK_041074
178	AT3G63030	SALK_042834
179	AT1G18835	SALK_009428
180	AT5G11060	SALK_020216
181	AT1G67120	SALK_057010
182	AT3G16980	SALK_032670
183	AT3G19670	SALK_066044C
184	AT3G19670	SALK_043590C
185	AT4G38710	SALK_062327C
186	AT1G11475	SALK_146725C
187	AT5G42950	SALK_059333C
188	AT1G14790	SALK_007638C
189	AT1G52520	SALK_019743C
190	AT2G17410	SALK_104394C
191	AT4G33620	SALK_090744C
192	AT4G33620	SALK_023493C
193	AT1G09730	SALK_080083C
194	AT3G24340	SALK_003876C
195	AT4G25180	SALK_073697
196	AT5G13730	SALK_027838C
197	AT4G25180	CS828668
198	AT1G09730	SALK_022079
199	AT1G80010	SALK_122261
200	AT1G14790	CS66077
201	AT4G33620	SALK_044756
202	AT5G24740	SALK_142760
203	AT2G04630	SALK_021069
204	AT5G12400	SALK_134905
205	AT1G55040	CS825466
206	AT1G55040	CS811560
207	AT2G17410	SALK_026835
209	AT4G19610	SALK_149991

208	AT1G16610	SALK_123442
210	AT5G41190	SALK_021098
211	AT2G30800	SALK_043338
212	AT5G20600	SALK_073773
213	AT1G56290	SALK_104857
214	AT3G43240	SALK_111627
215	AT3G29090	SALK_074820
216	AT3G06930	SALK_097442
217	AT4G38440	SALK_099872
218	AT1G15700	SALK_062789
219	AT2G34040	SALK_008073
220	AT2G39810	SALK_052108
221	AT3G42790	SALK_080056
222	AT3G12130	SALK_057095
223	AT5G49020	SALK_033423
224	AT4G20400	SALK_135712
225	AT4G00090	SALK_085277
226	AT4G14000	SALK_094629
227	AT1G24706	SALK_072011
228	AT1G60850	SALK_088247
229	AT2G38770	SALK_041627
230	AT5G64730	SALK_126634
231	AT5G51130	SALK_100446
232	AT3G63400	SALK_094094
233	AT5G20510	SALK_075676
234	AT5G05680	SALK_085349
235	AT1G06230	SALK_113292
236	AT2G34900	SALK_103499
237	AT4G21100	SALK_061944
238	AT5G14520	SALK_026359
239	AT1G73840	SALK_078793
240	AT1G79280	SALK_011827
241	AT2G33730	SALK_110742
242	AT2G24500	SALK_008119
243	AT5G06460	SALK_108047
244	AT5G15810	SALK_132252
245	AT4G16570	SALK_085829
246	AT5G51300	SALK_062177
247	AT2G41020	SALK_073780
248	AT4G24880	SALK_119615
249	AT5G62190	SALK_016729
250	AT5G03450	SALK_049477

251	AT1G11240	SALK_104924
252	AT1G03910	SALK_003714
253	AT5G13850	SALK_092796
254	AT5G58410	SALK_098351
255	AT3G51270	SALK_130093
256	AT5G42950	SALK_005994
257	AT2G17510	SALK_037533
258	AT1G14300	SALK_152562
259	AT1G71350	SALK_102168
260	AT5G64200	SALK_124563
261	AT4G31120	SALK_073624
262	AT5G06210	SALK_043816
263	AT5G12120	SALK_109895
264	AT5G47750	SALK_093225
265	AT4G02820	SALK_082825
266	AT2G38420	SALK_113273
267	AT5G20200	SALK_024526
268	AT5G47680	SALK_087697
269	AT2G25670	SALK_014065
270	AT3G12340	SALK_130890
271	AT2G40360	SALK_075405
272	AT1G07360	SALK_142085
273	AT1G50380	SALK_027789
274	AT3G20020	SALK_024873
275	AT5G53770	SALK_090264
276	AT1G48560	SALK_008422

Supplementary Table 2. Bs-sequencing basic information									
Sample Name	Read length(bp)	Clean Reads	Clean bases	Q20(%)	GC(%)	Depth(x)	Mapped Reads	Mapped ratio
Col-0	90	97,347,534	8,761,278,060	99.47;99.72	25.64	58	89,350,773	91.79%
clsy4-1	90	99,441,528	8,949,737,520	99.52;99.62	26.32	60	92,260,137	92.78%
clsy4-1/nrpd1-3	90	96,645,602	8,698,104,180	99.47;99.73	25.77	58	88,949,959	92.04%
clsy1-3	90	94,908,790	8,541,791,100	99.50;99.63	27.04	57	86,233,177	90.86%
clsy2-1	90	96,278,326	8,665,049,340	99.54;99.59	26.28	58	81,265,311	84.41%
clsy3-1	90	97,817,498	8,803,574,820	99.49;99.72	25.89	59	89,516,627	91.51%
clsy1-3/clsy2-1/clsy3-1	90	96,637,408	8,697,366,720	99.54;99.69	24.74	58	88,689,955	91.78%
clsy1-3/clsy3-1/clsy4-1	90	98,649,196	8,878,427,640	99.53;99.64	26.22	59	89,188,094	90.41%
clsy2-1/clsy3-1/clsy4-1	90	96,425,168	8,678,265,120	99.43;99.51	26.89	58	87,716,664	90.97%
clsy1-3/clsy2-1/clsy3-1/clsy4-1	90	97,113,818	8,740,243,620	99.54;99.65	26.61	58	90,014,686	92.69%

Supplementary Table 3. Mutants used in this study	
**Mutant name**	**T-DNA**
clsy1-3	
clsy2-1	**SALK_106745**
clsy3-1	**SALK_040366**
clsy4-1	**SALK_102252**
clsy4-2	**SALK_003876**
ros1-4	**SALK_045303**
nrpd1-3	**SALK_128428**
nrpe1-11	**SALK_029919**
dtf1-2	**SALK_074540**
clsy4nrpd1	***clsy4-1/nrpd1-3* (SALK_102252/SALK_128428)**
clsy4nrpe1	***clsy4-1/nrpe1-11* (SALK_102252/SALK_029919)**
clsy4ros1	***clsy4-1/ros1-4* (SALK_102252/SALK_029919)**
ros1nrpd1	***ros1-4/nrpe1-11* (SALK_045303/SALK_128428)**
ros1nrpe1	***ros1-4/nrpe1-11* (SALK_045303/SALK_029919)**
clsy12	***clsy1-3/clsy2-1* (*clsy1-3*/SALK_106745)**
clsy34	***clsy3-1/clsy4-1* (SALK_040366/SALK_102252)**
clsy123	***clsy1-3/clsy2-1/clsy3-1*(*clsy1-3*/SALK_106745/SALK_040366)**
clsy134	***clsy1-3/clsy3-1/clsy4-1*(*clsy1-3*/SALK_040366/SALK_102252)**
clsy234	***clsy2-1/clsy3-1/clsy4-1*(SALK_106745/SALK_040366/SALK_102252)**
clsy1234	***clsy1-3/clsy2-1/clsy3-1/clsy4-1*(*clsy1-3*/SALK_106745/SALK_040366/SALK_102252)**

Supplementary Table 4. Oligos used in this study

**Oligos Name**	**Oligos Sequence**	**Experiments**
*clsy1-3*F	GACCTATTGGGAATGTGGTTCAT	genotyping
*clsy1-3*R	CCGATTTCTTCATTCAATTCATAG	genotyping
*clsy2-1*F	TAGCCATGTATTTCCGGTGAG	genotyping
*clsy2-1*R	AGTGTTGTTGACCAGTGGGAG	genotyping
*clsy3-1*F	CTTCTTGCAGTGGCATTCTTC	genotyping
*clsy3-1*R	ATTAGGCGAAGAGGATGAAGC	genotyping
*clsy4-1*F	CTATGGAAACTCCTCCGTTCC	genotyping
*clsy4-1*R	TGATGCTTGTTCACCTTCTCC	genotyping
*clsy4-2*F	ACGACTTTGTCCTGGATGATG	genotyping
clsy4-2R	TTCGGATATGCGATGTTTCTC	genotyping
*nrpd1-3*F	GATCTGTTCAGCTTGCTCGTC	genotyping
*nrpd1-3*R	TTAATGTTCTTCATGCGGGAC	genotyping
*nrpe1-11*F	TTTTCCTCCAAGTTGTGATCG	genotyping
*nrpe1-11*R	AACAAGACCCAACGACTGTTG	genotyping
*ros1-4*F	CCAGTTAAGGACAGAACACCG	genotyping
*ros1-4*R	TCGTCTTTCGATCAAATCCAC	genotyping
LB1.3	ATTTTGCCGATTTCGGAAC	genotyping
AT5TE25535-RealF	GTCGTTGTTCCTTGAGTGTTTG	real-time PCR
AT5TE25535-RealR	CGGTTTCTCTCATACATCTCCTTT	real-time PCR
AtSN1-RealF	CCAGAAATTCATCTTCTTTGGAAAAG	real-time PCR
AtSN1-RealR	GCCCAGTGGTAAATCTCTCAGATAGA	real-time PCR
AT2TE07550-RealF	GTCCCTCGAGCAACAATACTAA	real-time PCR
AT2TE07550-RealR	ATGATGGATGGTAAGGCATAGAG	real-time PCR
AT4TE27915-RealF	GTCACGCACTCACCTTAACA	real-time PCR
AT4TE27915-RealR	GGACTTGTTGTAGGACGTTTCT	real-time PCR
AT5TE35950-RealF	CTAACGTCATTACATACACATCTTG	real-time PCR
AT5TE35950-RealR	TTGTTGATGTGAACTTTCATCC	real-time PCR
CLSY4-RealF	TGGGCCAGCAACATTAGGAACTCT	real-time PCR
CLSY4-RealR	TCCACTGATGCGGATGAAGTCGAT	real-time PCR
ROS1-RealF	AAGGACCAACTTGTTGCGAC	real-time PCR
ROS1-RealR	AGGACTCTATTAGCACTGAGC	real-time PCR
IGN5-RealF	AAGCCCAAACCATACACTAATAATCTAAT	real-time PCR
IGN5-RealR	CCGAATAACAGCAAGTCCTTTTAATA	real-time PCR
IGN6-RealF	TTTCGCCGTCACTAACATGTAATG	real-time PCR
IGN6-RealR	GAAGTAGCTTTTTCGGTCCAGTTC	real-time PCR
IGN20-RealF	AAGAACCGGACCAATACGG	real-time PCR
IGN20-RealR	CCACCGCCTCTATTGAAATG	real-time PCR
IGN22-RealF	TGGTCCATAGGTTCGGAATTT	real-time PCR
IGN22-RealR	GGCATGGTTTGATATCAGGAG	real-time PCR
IGN25-RealF	AAACCCACCTCTTTAGGTCCA	real-time PCR
IGN25-RealR	GGCTTGGAGAGTCCAACAAT	real-time PCR
IGN27-RealF	CGTTGTTCCGCCTAATTCTG	real-time PCR
IGN27-RealR	GCCAGGAAACCCTAACTTCC	real-time PCR
IGN29-RealF	CGTTTGTTTATGTAGGGCGAAAG	real-time PCR
IGN29-RealR	TAAAACTTTTCCCGCCAACCA	real-time PCR
IGN31-RealF	CAATCTGGCACACACGAAAC	real-time PCR
IGN31-RealR	CAGGTTGGATCTGTTGACGA	real-time PCR

Actin2-RealF	GAGAGATTCAGATGCCCAGAAGTC	real-time PCR
Actin2-RealR	TGGATTCCAGCAGCTTCCA	real-time PCR
At1g51170-RealF	TCGTCTCCTTCCTTCTCATCT	real-time PCR
At1g51170-RealR	ACTTTGAGTCTGTCGAGGTTAAG	real-time PCR
At1g63950-RealF	GATTGTGTGGATGGGTGTTTATG	real-time PCR
At1g63950-RealR	AGATGAGTGAATTCCGGGTAAG	real-time PCR
At1g65610-RealF	TAGCTGCTCTCGTTTCCTTAAC	real-time PCR
At1g65610-RealR	TCAAGGCTTCCAGGCTTTAG	real-time PCR
At2g27229-RealF	CATCTTCTAAATCGCCACTCCT	real-time PCR
At2g27229-RealR	TCCCTCACAATGATCTCTCTCT	real-time PCR
AT3g20690-RealF	TCTCTCTAGGCTTCCGTTGATA	real-time PCR
AT3g20690-RealR	TACATTGCCGATGTGCTTATTTG	real-time PCR
AT3g25670RealF	GGAGAAGGCAGAGGATAGTTTG	real-time PCR
AT3g25670RealR	CTAGTAACCATCACACCCATTGA	real-time PCR
At3g47540RealF	TGCCTCGTAGGTCCTTGTATAG	real-time PCR
At3g47540RealR	CCATTACCGGCTTGGTTGATAA	real-time PCR
At5g39550RealF	TCCAAGACCGTTGCCTAATG	real-time PCR
At5g39550RealR	GACCCTCAGCTTCATCGAAAT	real-time PCR
AT5g44910RealF	CGAGGAACTGAGGGAGAAATAC	real-time PCR
AT5g44910RealR	TGCCCTCACAAGACCTAAAC	real-time PCR
At2g45380RealF	CAACTCAAGTGGTGGTGTAGAG	real-time PCR
At2g45380RealR	GAATTGCCTTTCCATGCTTCTT	real-time PCR
At4g11790RealF	ACGGCCATCCAAGAATACG	real-time PCR
At4g11790RealR	AGAGAGAGGACGACTCGATAAG	real-time PCR
AT5g39850RealF	AAGCCACGTCGTCCTTATG	real-time PCR
AT5g39850RealR	TTGAACTCTCCAAAGCTCTCTC	real-time PCR
PM36-ChIP-1F	CACATAACCGGTGGAGGATTT	ChIP assay
PM36-ChIP-1R	GTAGCTCGATCCAAGAGCTTTC	ChIP assay
PM36-ChIP-2F	GTGTCAAGTAACCCGAGAGAG	ChIP assay
PM36-ChIP-2R	GATCTTAGCTTCATCCACGTTTG	ChIP assay
PM36-ChIP-3F	ACCACTCGGATTGATTCCAATA	ChIP assay
PM36-ChIP-3R	GACGCAAAGGAAACAGAGAATAAG	ChIP assay
PM36-ChIP-4F	GTAAGGTCCCGCAACTTCATAA	ChIP assay
PM36-ChIP-4R	CCGGAAAGGAAACTTGTTCAATC	ChIP assay
PM27-ChIP-1F	ATTACACGAACCGGAGGAATG	ChIP assay
PM27-ChIP-1R	GGGAATTTGAAGAGATTGATGGATG	ChIP assay
PM27-ChIP-2F	TTTACACGAACCGGAGGAATAC	ChIP assay
PM27-ChIP-2R	GGTGAGACAAACGTGGATGA	ChIP assay
ID2-ChIP-1F	AGGCATGTTTCCATGTTTGATG	ChIP assay
ID2-ChIP-1R	TCCAGTGATGACAGGATCTTATTG	ChIP assay
ID2-ChIP-2F	GCTGCGAGGAACTGAAATTG	ChIP assay
ID2-ChIP-2R	TGCTCTGGTTGGTATGTGTATAA	ChIP assay
ID19-ChIP-1F	CTATCGGCTGATATGGTCAGTTT	ChIP assay
ID19-ChIP-1R	TGACCTCCTGACAAACATTCC	ChIP assay
ID19-ChIP-2F	TCTCTAAAGGGCCACCATACT	ChIP assay
ID19-ChIP-2R	ACTTGGACCAAAGGCTCAAC	ChIP assay
clsy4-hyperDMR-1F	CCAAACGCCACAGAGAGTAA	ChIP assay
clsy4-hyperDMR-1R	CGCTTGGATTGCTTCCAATATC	ChIP assay
clsy4-hyperDMR-2F	ACTAAACTCTACAGAATTTGTTGGC	ChIP assay

clsy4-hyperDMR-2R	GGTAATATTGGGTTAGTCTAGGTGAT	ChIP assay
clsy4-hyperDMR-3F	TCTCCGCTTCTCTATTCCAAAC	ChIP assay
clsy4-hyperDMR-3R	AGGGACACTCAACAACTTCAT	ChIP assay
clsy4-hyperDMR-4F	GGGCTACTTGTTGTATACCTTGA	ChIP assay
clsy4-hyperDMR-4R	TCAGACAATCAGAACCCGAAATA	ChIP assay
clsy4-hyperDMR-5F	CATCGGACATACAAACCTGAAAC	ChIP assay
clsy4-hyperDMR-5R	CTGGAGAAGCTGAAACACTTTG	ChIP assay
clsy4-hyperDMR-6F	ACACATAGTCTCTTCGCTTGAC	ChIP assay
clsy4-hyperDMR-6R	CCGAGCTGCTTGATGACTT	ChIP assay
clsy4-hyperDMR-7F	CCTTCTGATGAGTGTTAGGTTCT	ChIP assay
clsy4-hyperDMR-7R	TCAATTGGTAATAGGTGGAGTGA	ChIP assay
clsy4-hyperDMR-8F	AAAGCTCCGATGTTTGGATAGA	ChIP assay
clsy4-hyperDMR-8R	CACATGATTTGGTGGAGGAGA	ChIP assay
clsy4-hyperDMR-9F	TAGAGCAACTCCAACCTTGTATC	ChIP assay
clsy4-hyperDMR-9R	GCTAAGGCTTGTTGGTTTCATT	ChIP assay
clsy4-hyperDMR-10F	CAGTCAAGCTTCCTTGTTCATTAC	ChIP assay
clsy4-hyperDMR-10R	ATGCATGGTTGCCCATCT	ChIP assay
Tublin8-RealF	ATAACCGTTTCAAATTCTCTCTCTC	ChIP assay
Tublin8-RealR	TGCAAATCGTTCTCTCCTTG	ChIP assay
ros1-hyperDMR-1F	CCAAGAAGCCAAGCAGGAAA	ChIP assay
ros1-hyperDMR-1R	TGCTTGAATCGGTGTCTTCG	ChIP assay
ros1-hyperDMR-2F	ACAGTGTTCGTAAAGATGTAGCC	ChIP assay
ros1-hyperDMR-2R	AGCACATCACAATGAACGTA	ChIP assay
ros1-hyperDMR-3F	AACTCAGGCAACGGTATGGT	ChIP assay
ros1-hyperDMR-3R	CCGCGTCGAGGAAATAAGTC	ChIP assay
ros1-hyperDMR-4F	TGGTCCAAATTATTTCTCCAAAGACA	ChIP assay
ros1-hyperDMR-4R	TATATCAGGCGGGAGCGTAG	ChIP assay
DTF1-ChIP-1F	GATCTACCAACGAACCTCCATC	ChIP assay
DTF1-ChIP-1R	TCCTCCTTCTTTGTGGCTTAAT	ChIP assay
DTF1-ChIP-2F	CGCCTACCTCTGTACCTTCTTA	ChIP assay
DTF1-ChIP-2R	CTGGTGGATTTCTTCGTCAGTC	ChIP assay
DTF1-ChIP-3F	TAGCTAATAACATAAGAGTGCACCA	ChIP assay
DTF1-ChIP-3R	AAACCGACTATCGTTTCTTATCTCT	ChIP assay
DTF1-ChIP-4F	CCCATATTGAAACGGTGGATTA	ChIP assay
DTF1-ChIP-4R	TGGTGCACTCTTATGTTATTAGC	ChIP assay
DTF1-ChIP-5F	CACGTGGAATTCAGTGGATGA	ChIP assay
DTF1-ChIP-5R	CGTCCTGTATCATCACATCAGC	ChIP assay
DTF1-ChIP-6F	TGTTATTAAGTAACGTCATTCCAAACCC	ChIP assay
DTF1-ChIP-6R	ATTAGCGGTGTACTGCGGTT	ChIP assay
DTF1-ChIP-7F	GGCTTATTGTACATTATCTCGATCTC	ChIP assay
DTF1-ChIP-7R	TGTTACCGACAATACCCTAACC	ChIP assay
IGN5-ChopF	TCCCGAGAAGAGTAGAACAAATGCTAAAA	Chop-PCR
IGN5-ChopR	CTGAGGTATTCCATAGCCCCTGATCC	Chop-PCR
Solo-LTR-ChopF	ATTAACCACACGCTGCATCTAAT	Chop-PCR
Solo-LTR-ChopR	GAGAGACGATCGTGCTGATAAC	Chop-PCR
AtSN1-ChopF	ACTTAATTAGCACTCAAATTAAACAAAATAAGT	Chop-PCR
AtSN1-ChopR	TTTAAACATAAGAAGAAGTTCCTTTTTCATCTAC	Chop-PCR

PM36-ChopF	TGACCTGCATAGGCTATAACACA	Chop-PCR
PM36-ChopR	ATTGGAATCAATCCGAGTGG	Chop-PCR
PM27-ChopF	ATAGATCCTAATAGAGGCGTAA	Chop-PCR
PM27-ChopR	AGAATAACAAATGGCACAGA	Chop-PCR
Pm36-Bis-F	GTAGTTTGAGATGATTAATGATAGAGTT	Bisulphite sequencing
Pm36-Bis-R	AAACTTATTCAATCTTCAATACTCTAC	Bisulphite sequencing
Pm27-Bis-F	GATTATTTAGTYGGTATTTYGAYGAGT	Bisulphite sequencing
Pm27-Bis-R	CCAAAAAATACTTAAATCCATAAATCTTAA	Bisulphite sequencing
ID2-Bis-F	AGGGGTAAAAATGAGAGGATTAATTTA	Bisulphite sequencing
ID2-Bis-R	CTCTTACCTCTAAATTATATAACATT	Bisulphite sequencing
ID19-Bis-F	GATAGAAATGTATGAATTTTTTTAGAG	Bisulphite sequencing
ID19-Bis-R	TCACAAAATAACTACAAAAATATATAT	Bisulphite sequencing
ID30-Bis-F	GTATGTTTTTTTATATATAAAAGAGAGT	Bisulphite sequencing
ID30-Bis-R	ATCTTATCCTCATTAATCACACATAACA	Bisulphite sequencing
CLSY4-F	ATGAATTCGAAGCTAAAATCAAGCAAAAAACG	Gene cloning
CLSY4-R	TATCTAGAGAAGAAGCTGGTATTCATGTCAG	Gene cloning
Pm36-probeF	TGACCTGCATAGGCTATAACACA	Northern blotting
Pm36-probeR	ATTGGAATCAATCCGAGTGG	Northern blotting
ID2-probeF	AAGATCCTGTCATCACTGGAAAA	Northern blotting
ID2-probeR	TGGTGACAGTGCTTGTGGTT	Northern blotting
ID19-probeF	TTCTCTCTAAAGGGCCACCA	Northern blotting
ID19-probeR	TCTATCCGAACATTGGCACA	Northern blotting
miR171-probe	GATATTGGCGCGGCTCAATCA	Northern blotting
DD1-probeF	CGGGATCGATAGGATCTTCA	Northern blotting
DD1-probeR	GTTTTGCCTCTTTGGTGGTC	Northern blotting
DD2-probeF	CCTTTCTCTTGTTCGCCCTA	Northern blotting
DD2-probeR	CAAGGTTCGGGAAAAGATCA	Northern blotting
DD3-probeF	CTCTCCACATGTTTTGGGTTA	Northern blotting
DD3-probeR	TTCCAAGATGAGAACTGCACA	Northern blotting
DD12-probeF	CGGTGATGCAAACGGATAG	Northern blotting
DD12-probeR	TGGCAAAACTAACGCACAAA	Northern blotting
miR167-probe	TAGATCATGCTGGCAGCTTCA	Northern blotting
